# Determinants of Human Cyclin B1 Association with Mitotic Chromosomes

**DOI:** 10.1371/journal.pone.0059169

**Published:** 2013-03-11

**Authors:** Kathleen L. Pfaff, Randall W. King

**Affiliations:** Harvard Medical School Department of Cell Biology, Boston, Massachusetts, United States of America; Mayo Clinic, United States of America

## Abstract

Cyclin B1–CDK1 activity is essential for mitotic entry, but questions remain regarding how the activity of this kinase is spatially regulated. Previous studies showed that the cyclin B1 subunit localizes to several compartments of a mitotic cell, including the centrosomes, mitotic spindle, kinetochores and chromosomes via distinct sequence elements. Mitotic chromosome association occurs through the unstructured N-terminal domain of cyclin B1 and is independent of CDK1 binding. Here, we use live cell imaging of human cyclin B1 fused to GFP to precisely define the sequence elements within cyclin B1 that mediate its association with condensed mitotic chromosomes. We find that a short, evolutionarily conserved N-terminal motif is required for cyclin B1 to localize to mitotic chromosomes. We further reveal a role for arginine residues within and near the destruction box sequence in the chromosome association of cyclin B1. Additionally, our data suggest that sequences further downstream in cyclin B1, such as the cytoplasmic retention sequence and the cyclin box, may negatively modulate chromosome association. Because multiple basic residues are required for cyclin B1 association with mitotic chromosomes, electrostatic interactions with DNA may facilitate cyclin B1 localization to chromosomes.

## Introduction

Protein phosphorylation catalyzed by mitotic protein kinases drives the profound changes in cell morphology and function that define mitosis [1], [2]. Cells prepare for chromosome segregation and cell division by dissolving the nuclear envelope, condensing chromosomes, and building a bipolar mitotic spindle. Biochemical processes such as cap-dependent translation and transcription are inactivated [3], [4]. The proper execution of these mitotic events requires the appropriately timed activation of mitotic protein kinases, including cyclin-dependent kinases, aurora kinases, and polo-like kinases, among others [1], [5]. In addition to precise temporal control of kinase activity, proper localization of these kinases in specific regions of the dividing cell is critical for the kinases to function appropriately. By localizing to distinct sub-compartments of the cell, protein kinases can accentuate phosphorylation of subpopulations of substrates, providing another layer of specificity in mitotic control. Determining how protein kinases are localized during mitosis is therefore important for understanding how mitosis is regulated.

Mitotic protein kinases use protein-protein interaction motifs, independent of the catalytic domain, to localize the kinase to particular substrates or regions of the cell. For example, Polo-like kinases utilize a polo box domain (PBD) to recognize substrates that have been previously phosphorylated by CDKs [6], providing a functional and spatial coupling between Plk1 and CDK activity. In the case of cyclin-dependent kinases, the cyclin subunit plays a critical role in activating the catalytic domain, but also confers substrate specificity and dictates localization of the CDK. During interphase, cyclin A is predominantly nuclear whereas B-type cyclins are cytoplasmic [7], [8]. At the onset of mitosis, cyclin B1 translocates to the nucleus prior to nuclear envelope breakdown, and associates with centrosomes, the mitotic spindle, condensing chromosomes and unattached kinetochores during mitosis [7], [9], [10], [11]. Distinct sequence elements in cyclin B1 appear to mediate interaction with these various structures within the mitotic cell. For example the MRAIL sequence in cyclin B1 is important for centrosome association, whereas the N-terminal unstructured domain is important for association with mitotic chromosomes [9].

Localization of CDK activity to mitotic chromosomes is likely to be important for proper execution of mitosis, as several CDK substrates are localized to mitotic chromosomes. CDK1-dependent phosphorylation promotes the association of proteins such as RCC1 with mitotic chromosomes [12], [13]. Binding of RCC1 to mitotic chromosomes is important for spindle assembly by maintaining a gradient of activated Ran [14], [15], [16]. Phosphorylation of RCC1 inactivates its binding to importins, which otherwise antagonize the ability of RCC1 to bind to chromosomes [12], [13]. As another example, CDK1-dependent phosphorylation of condensin is required for timely chromosome condensation [17], [18]. In this context, phosphorylation regulates condensin's supercoiling activity rather than its association with the chromosomes. Furthermore, CDK1 activity also promotes chromosome condensation by phosphorylating and ejecting the histone demethylase PHF8, resulting in the accumulation of monomethylated histone H4K20 that promotes condensin binding [19]. Finally, CDK1 is responsible for phosphorylating and inhibiting separase, which localizes to mitotic chromosomes [20], [21], [22], [23], [24]. Direct inhibition of separase by cyclin B1/CDK1 may play a critical role in ensuring faithful timing of the initiation of chromosome segregation during mitosis [22]. Together these studies indicate that the localization and activity of several of the critical regulators of chromosome condensation and mitotic spindle formation may depend on the ability of cyclin B1 to promote CDK1 interaction with mitotic chromosomes.

Here we utilize time-lapse imaging to characterize sequence determinants required for association of cyclin B1 with mitotic chromosomes. We define the minimal protein sequence that is sufficient for chromosome association, and we identify a short, conserved basic sequence element that is critical for association of full-length cyclin B1 with mitotic chromosomes. We also explore the role of the destruction box in association with mitotic chromosomes and provide evidence that this region is important in promoting chromosome association. Our results are consistent with a model in which electrostatic interactions between basic residues in the cyclin B1 N-terminus facilitate interaction with chromatin, although we cannot rule out a model in which the specific conserved residues interact with specific receptor proteins on mitotic chromosomes.

## Materials and Methods

### Cell culture and creation of stable cell lines

BS-C-1 and HeLa cells (American Type Culture Collection, Manassas, VA) were cultured in DMEM (Mediatech, Manassas, VA) supplemented with 10% FBS (Atlanta Biologicals, Lawrenceville, GA) in T75 flasks and maintained in a humidified 37°C incubator supplied with 5% CO_2_ (Thermo). To make stably expressing HeLa cell lines, the WT^1–41^-GFP or Δ3–8^1–41^-GFP fusions and GFP alone were cloned into the pBabe puro vector (a gift from N.Solimini and S. Elledge). Retrovirus was generated by co-transfecting 293T cells (a gift from N. Solimini and S. Elledge, ATCC) with the pBabe vector, pCG gag-pol and pVSV-G using Fugene 6 (Roche, Indianapolis, IN) for 24 hours. HeLa cells were exposed to retrovirus for 24 hours and selected using 0.25 µg/mL puromycin (Sigma) for 3 days.

### Adenoviral expression in hela cells

The generation of full-length human cyclin B1-GFP adenovirus was previously described [9]. HeLa cells were exposed to a 1∶500 dilution of purified adenovirus for 48 hours before collection for lysis and fractionation.

### Plasmids and transfection

For expression in BS-C-1 cells, all cyclin B1 fragments were cloned in the pEGFPN1 vector (Clontech, Mountain View, CA). Truncated cyclin B1 pieces were generated by PCR amplification. WT^1–15^ and WT^1–20^ fragments were ordered as complementary oligonucleotide sequences with restriction site overhangs (IDT, Coralville, IA), annealed, and cloned into the pEGFPN1 vector. N-terminal mutagenesis was performed using mutagenic forward primers in a PCR amplification reaction. All other mutations were introduced using the Quick Change Site-Directed Mutagenesis Kit (Agilent, Santa Clara, CA).

### Fluorescence live cell imaging

BS-C-1 cells were plated in 35 mm glass-bottom petri dishes (MatTek Corp, Ashland, MA) 18–24 hours prior to plasmid transfection using Fugene 6 (Roche, Indianapolis, IN). Twenty-four hours after transfection, the dishes were inserted into a covered chamber supplied with 5% CO_2_ and mounted onto a linearly encoded motorized microscope stage (Prior Scientific, Rockland, MA). DIC and FITC images were captured at 10 minute intervals for 24–48 hours using a Nikon Ti inverted fluorescence microscope fitted with a 37°C enclosed incubation chamber and using a 40X Plan Fluor 0.75 NA objective lens. A Hamamatsu ORCA cooled CCD camera collected the images with 2X2 binning using Nikon Elements software (version 3.0). For the qualitative assay, movies were manually analyzed using Nikon Elements software. All cells expressing GFP reporter that underwent mitosis during the duration of the experiment were recorded for presence or absence of chromosome-associated signal in metaphase. Because not all cells could be categorized, a third category (“cannot call”) was utilized to describe cells that showed strong overexpression or underexpression of the GFP reporter or did not have a well-defined metaphase plate by DIC optics (examples, Figure S1A). For most constructs, 20–30% of the expressing mitotic cells were scored as “cannot call”. For presentation of representative images, the ND files were exported as TIFF files and light intensity levels were adjusted using the same conditions for all images with Metamorph Image Analysis Software (Molecular Devices, Sunnyvale, CA).

### Fluorescence intensity quantitation

Fluorescence intensities of metaphase cells were measured using Nikon Elements software (version 3.1). The first captured frame of metaphase was always selected for measurement. A region corresponding to the chromosomes of the metaphase plate was hand-selected in the DIC channel. The mean fluorescence intensity for the corresponding region of the FITC channel was calculated. The region of the whole cell was determined by a threshold set at 12 standard deviations above the average background signal in the FITC channel, and the whole mean cell fluorescence intensity was calculated. The chromosome enrichment ratio (CER) was determined by dividing the background-subtracted chromosome mean intensity by the background-subtracted whole cell mean intensity. At least 8 cells per construct were measured and the results plotted as box-and-whisker diagrams. All cells chosen for this quantitative assay had a well-defined metaphase plate and a mean whole cell fluorescence intensity between 50 and 500.

### Statistics

For the qualitative analysis, a Fisher's exact test was employed using JMP software for statistical analysis to compare the frequency of cells that were scored for positive chromosome association. A 2-variable analysis was carried out to compare the frequency of cells that exhibited positive chromosome association versus the combined frequencies of cells that lacked chromosome association and those that were scored as cannot call (Table S1). For the quantitative analysis of chromosome localization, the Anderson-Darling test was used to determine whether or not the data had a normal distribution. Most of the constructs revealed a normal distribution, although a couple, including WT^1–433^, did not have a normal distribution. Thus, the statistical significance of differences in the CER among various constructs was determined with the Wilcoxon Exact Test using JMP software (Table S2).

### Mitotic cell collection

1×10^6^ parental HeLa cells or derivatives stably expressing a GFP fusion protein were plated in T75 flasks and expanded for 3 days before adding 330 nM nocodazole (Sigma) for 18 hours. Cells were collected by mitotic shakeoff, washed once in PBS (Dulbecco's, Corning), and frozen in liquid nitrogen.

### HeLa cell fractionation

The mitotic cell fractionation protocol was adapted from Abe et al., 2011 [17]. Mitotic HeLa cell pellets (containing approximately 2×10^6^ mitotic cells) were thawed slowly on ice for 25 minutes and resuspended in 100 µL PBS. 150 µL MilliQ H_2_0 was added and hypotonic lysis was carried out on ice for 5 min. Whole cell extract samples were set aside. The remaining sample was centrifuged at 5000 rpm for 5 min at 4°C. The cytoplasmic supernatant was collected and the chromosome-enriched pellet was resuspended in IP lysis buffer (75 mM HEPES pH 7.4, 1.5 mM EGTA, 1.5 mM MgCl_2_, 150 mM KCl, 15% glycerol, 0.075% NP40). Pierce BCA Protein Assay Kit (Thermo) was used to determine the protein concentration for all samples.

### Immunoblotting and quantitatation

12 µg of total protein lysate was loaded onto 4–12% Bis Tris gradient gels (Invitrogen) and resolved by SDS-PAGE. Samples were transferred to PVDF using a wet transfer apparatus (Bio-Rad). Primary antibodies against cdc2 (Santa Cruz, sc-54, 1∶500), cyclin B1 (Santa Cruz, sc-245, 1∶750), GFP (Santa Cruz, sc-8334, 1∶500), Histone H2B (Abcam, ab1790, 1∶1000), and α-tubulin (Sigma, T9026, 1∶500) were used at indicated dilutions. HRP-conjugated anti-rabbit or anti-mouse antibodies (GE Healthcare) were used at a 1∶5000 dilution. SuperSignal West Pico Chemiluminescent Substrate (Thermo) was used and detected using a LAS3000 Luminescent Image Analyzer (Fuji). The gel analysis package in ImageJ was used for quantitation of signal on immunoblots. The percent of protein associated with the chromosome-enriched fraction was calculated as the value of the chromosome fraction divided by the sum of the chromosome and cytoplasmic fractions. The protein abundance of adenovirally expressed cyclin B1-GFP in BS-C-1 cells or the stable WT^1–41^-GFP and Δ3–8^1–41^-GFP transgenes in HeLa cells was compared to endogenous cyclin B1 by blotting both samples with the cyclin B1 antibody and then comparing the values of the bands for the mitotic whole cell extract (WCE) lane.

### Protein alignment

The PRALINE multiple sequence alignment tool (available at http://www.ibi.vu.nl/programs/pralinewww/) was used with standard settings to produce the protein sequence alignment of the first 50 amino acids of cyclin B1 from selected species.

## Results

### Endogenous cyclin B1 and CDK1 fractionate with mitotic chromosomes

**Figure 1:**
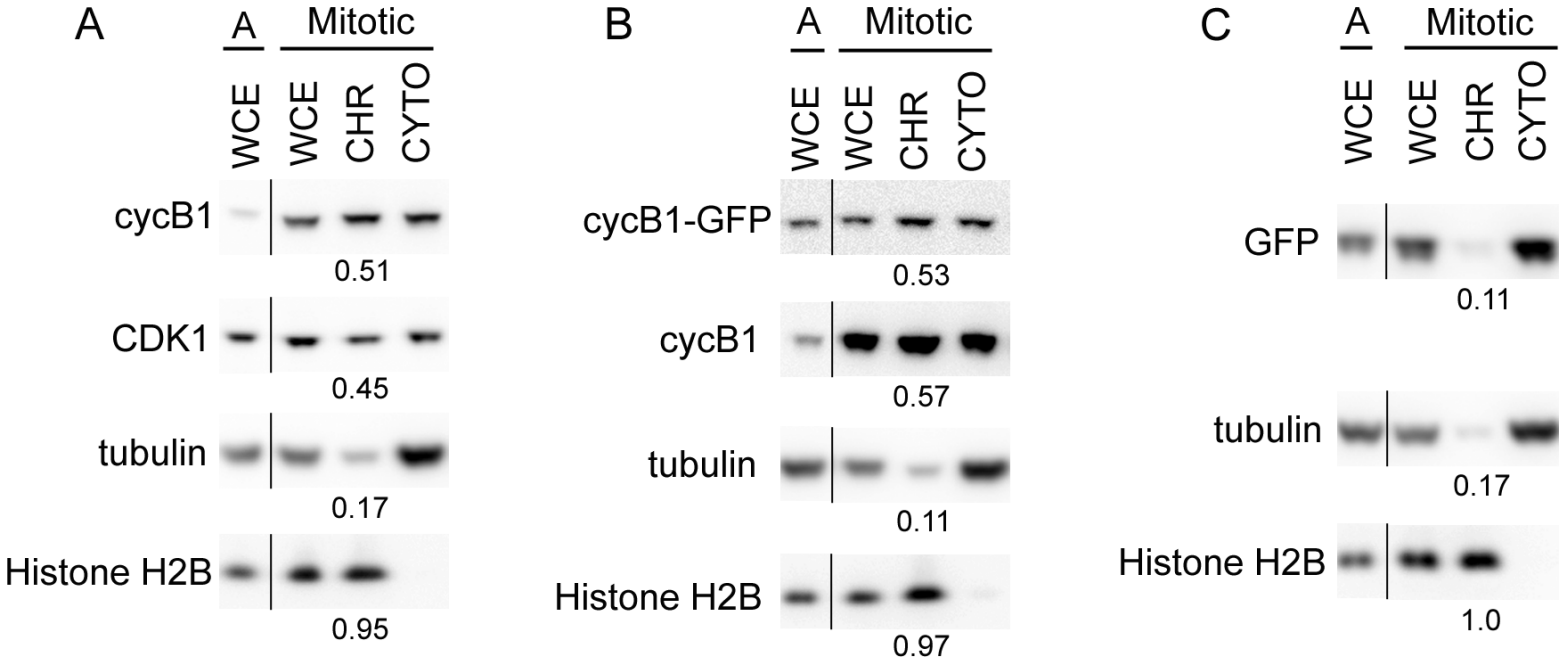
Biochemical fractionation of HeLa cells demonstrates that cyclin B1 and CDK1 associate with mitotic chromosomes. **A**. Endogenous cyclin B1 and CDK1 associate with a chromosome-enriched fraction. Hypotonic lysis of asynchronous (A) or mitotic HeLa cells produced whole cell extract (WCE), followed by fractionation of the mitotic lysate into chromosomal (CHR) and cytoplasmic fractions (CYTO). Western blotting was visualized with a Fuji LAS3000 Image Analyzer and quantified with the gel analysis package on ImageJ. The numbers below the CHR bands are the fraction of the total protein (CHR+CYTO) in the CHR fraction. α-Tubulin was used as a cytoplasmic control and indicated there was some contamination of cytoplasmic material in the chromosomal fraction. Histone H2B was used as the chromosomal control. **B**. Adenovirally expressed full length cyclin B1-GFP associates with mitotic chromosome-enriched fraction of HeLa cells. Experimental procedure and abbreviations as in part A. Cyclin B1-GFP was detected with the cyclin B1 antibody. **C**. GFP is not present in the chromosome-enriched fraction of mitotic HeLa cells that stably express GFP. Experimental procedure and abbreviations as in part A.

Several lines of evidence have shown that the cyclin B1/CDK1 complex associates with chromosomes during mitosis [7], [9], [10], [11], [25], [26], [27]. To more quantitatively determine the fraction of cyclin B1 and CDK1 that associates with chromosomes, and to determine whether GFP-tagged cyclin B1 recapitulates this behavior, we fractionated mitotic cell lysates into chromosome-enriched and cytosolic fractions using a previously established method [17]. The relative amounts of endogenous cyclin B1 and CDK1 in the mitotic chromosome fraction were determined by quantitative Western blotting. Blotting with a histone H2B antibody indicated that there was minimal contamination of chromosomal proteins in the cytoplasmic fraction (Figure 1A). As expected, there was some contamination of cytoplasmic proteins in the chromosome-enriched fraction; blotting with a tubulin antibody indicated that about 17% of tubulin was present in the chromosomal fraction (Figure 1A). We found that 51% of total cellular cyclin B1 was present in the chromosome-enriched fraction of mitotic cells (Figure 1A). Similarly, 45% of total cellular CDK1 was present in the chromosome fraction (Figure 1A). Given the presence of 17% of cytoplasmic protein in the chromosomal fraction, our data indicate that approximately 30–40% of endogenous cyclin B1 and CDK1 are associated with mitotic chromosomes. We used the same fractionation approach to examine the chromosome association of adenovirally expressed cyclin B1-GFP. The exogenous cyclin B1-GFP was expressed at about half the level of endogenous cyclin B1 in mitotic cells (Figure 1B). Similar to endogenous cyclin B1, 53% of cyclin B1-GFP protein was associated with the chromosome-enriched fraction (Figure 1B). In contrast, only a small proportion of GFP protein (11%) from HeLa cells stably expressing GFP was found in the mitotic chromosome-enriched fraction (Figure 1C), comparable to the degree of cytoplasmic contamination of the chromosome-enriched fraction as seen with tubulin (Figure 1A–1C). Together these results indicate that endogenous cyclin B1 and CDK1 are present in the chromosome-associated fraction and that exogenously expressed cyclin B1-GFP recapitulates this association.

### Mapping of sequence determinants that are necessary and sufficient for cyclin B1 association with mitotic chromosomes

**Figure 2:**
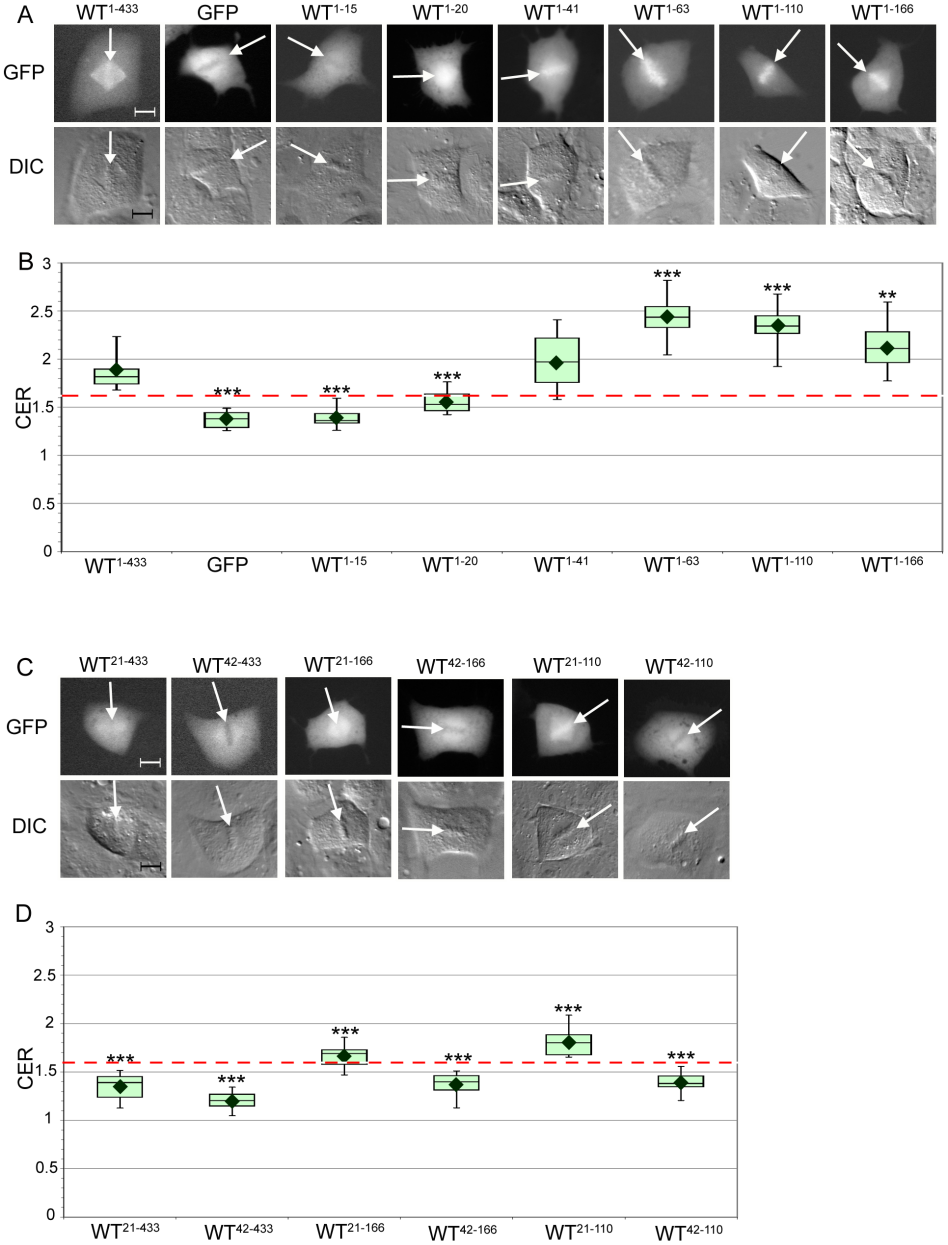
The first 20 amino acids of cyclin B1 can promote association with mitotic chromosomes. **A**. Representative images of mitotic BS-C-1 cells expressing GFP fusions of WT^1–433^ cyclin B1, GFP only, or fragments of cyclin B1 that lack the C-terminus. The GFP signal is detected by the FITC channel (top) and the position of the metaphase plate (indicated with white arrows) can be identified with DIC optics (bottom). Scale bar  =  10 µm. **B**. Box-and-whisker plots of Chromosome Enrichment Ratios (CER) for the constructs shown in part A. The CER is calculated as the ratio of the mean fluorescence intensity of the chromosomal region to the mean fluorescence intensity of the entire cell. The diamond indicates the mean CER. The box represents the 2^nd^ and 3^rd^ quartiles of the data, with the horizontal line representing the median and the whiskers representing the range. The red dotted line indicates the threshold of 1.6 that correlates with chromosome association in the qualitative assay. N = 8–14, ** indicates p < 0.01 and *** indicates p<0.001 compared to WT^1–433^. Further details including mean CER, standard deviations, and Wilcoxon exact test p-values can be found in Table S2. **C**. Representative mitotic BS-C-1 cells expressing GFP fusions of cyclin B1 fragments that lack different regions of the N- and C-termini. The metaphase plate is indicated by white arrows. Scale bar = 10 µm. **D** CER plots for the constructs shown in part C. The red dotted line indicates the threshold of 1.6. N = 10–11, *** indicates p<0.001 compared to the WT^1–433^, WT^1–166^, WT^1–110^, respectively. Further details including mean CER, standard deviations, and Wilcoxon exact test p-values can be found in Table S2.

To identify sequences in human cyclin B1 that promote its association with mitotic chromosomes, we imaged BS-C-1 cells expressing various fragments of cyclin B1 fused to GFP. Since these cells express endogenous cyclin B1, the exogenous cyclin B1-GFP must compete for localization at all structures. During metaphase, full-length cyclin B1-GFP (WT^1–433^) was found to associate with the centrosomes, mitotic spindle and chromosomes (Figure 2A), as previously reported [9], [11], [28], [29], [30]. Kinetochore association was not assessed in the present study because low-magnification wide-field imaging was employed, which cannot discern kinetochore localization [9]. Utilizing a qualitative approach to ascertain the presence or absence of fluorescent signal on the metaphase plate, we found that the majority (61%) of mitotic cells expressing WT FL cyclin B1-GFP showed association of the fluorescent signal with the metaphase plate (Figure S1B). A small fraction (2%) of the mitotic population expressing WT^1–433^ appeared to lack cyclin B1-GFP signal on the mitotic chromatin, which may be a consequence of competition with endogenous protein. GFP itself was strongly excluded from condensed mitotic chromosomes, localizing to the cytoplasm of mitotic cells with enhanced signal around the mitotic spindle (Figure 2A). In both instances, there was a subset of cells (25–35%) for which mitotic chromatin association could not be determined by this qualitative assay (Figure S1B). We therefore developed a quantitative approach to measure the intensity of cyclin B1-GFP fluorescence associated with mitotic chromosomes. We defined a Chromosome Enrichment Ratio (CER) as the ratio of the mean fluorescence intensity of the chromosomal signal to the mean whole cell intensity. WT^1–433^ had a mean CER of 1.87 whereas GFP alone had a mean CER of 1.37, indicating that cyclin B1 can promote localization of GFP to chromosomes (Figure 2B, Table S2, p<0.0001). The fact that the CER for GFP is greater than one is likely a consequence of the fact that the metaphase plate is present in the thickest part of the cell, where there is fluorescent protein present in the cytoplasm and associated with the neighboring mitotic spindle. In subsequent experiments examining cyclin B1-GFP mutants, we found that a CER above 1.6–1.7 correlated with positive chromosome association as measured by our qualitative assay.

We previously reported that the N-terminal 109 amino acids of cyclin B1 are necessary and sufficient to localize GFP to mitotic chromosomes [9]. To identify the minimum sequence sufficient for association with mitotic chromosomes, we created a series of cyclin B1 truncations fused to GFP and used live-cell imaging, combined with the qualitative and quantitative analyses, to assess their localization. The first 15 amino acids of cyclin B1 (WT^1–15^) did not promote GFP association with mitotic chromosomes (Figure 2A, 2B, S1B) and had a mean CER of 1.39, similar to that of GFP alone (1.37). In contrast, fusion of the first 20 amino acids of cyclin B1 (WT^1–20^) to GFP promoted weak localization to mitotic chromosomes, increasing the mean CER to 1.55 (Figure 2A, 2B, S1B, p = 0.002 compared to GFP alone). However, this value was significantly lower than the mean CER of 1.87 for the full-length protein WT^1–433^ (p<0.001, Table S2). Furthermore, the chromosome association of WT^1–20^ was often a blurred fluorescence signal surrounding the metaphase plate and the mitotic spindle. These findings suggest that the N-terminal twenty amino acids of cyclin B1 contains a sequence that promotes chromosome association, but that other regions of the protein also contribute to chromosome localization.

Downstream of this region, the cyclin B1 protein has several important functional motifs including the destruction box (D-box; amino acids 42–50) [31], the cytoplasmic retention sequence (CRS, amino acids 110–166) [8], and the cyclin box (amino acids 199–310) [32], which binds CDK1. We created a series of GFP fusion proteins based on the arrangement of these functional motifs (summarized in Figure S2A). We examined localization of fusion proteins encoding the first 41 amino acids (WT^1–41^, includes sequence N-terminal to the D-box); the first 63 amino acids (WT^1–63^, includes the D-box), the first 110 amino acids (WT^1–110^, includes sequence N-terminal to the CRS) and the first 166 amino acids (WT^1–166^, includes the CRS but lacks the CDK1 binding domain) of cyclin B1. In interphase cells, we observed that WT^1–41^, WT^1–63^, and WT^1–110^ were present in both the nucleus and the cytoplasm, whereas WT^1–166^ was exclusively cytoplasmic, consistent with the presence of the CRS region (Figure S2B). In metaphase, each of these cyclin B1 fragments fused to GFP exhibited association with mitotic chromosomes (Figure 2A, 2B, S1B). WT^1–41^ had a mean CER of 1.97, which was significantly increased compared to WT^1–20^ (p = 0.002), but was not significantly different from WT^1–433^ (Figure 2B, Table S2). Extension of the N-terminal region to include 63 amino acids (WT^1–63^) increased the CER to 2.45, which was significantly greater than WT^1–433^ (p<0.001), suggesting the presence of an additional element between amino acids 41 and 63 that promotes chromosome association (Figure 2B). Expression of a cyclin B1 fragment encoding the first 110 amino acids (WT^1–110^) yielded a CER of 2.34, which remained statistically significantly increased compared to WT^1–433^ (p<0.001, Figure 2B). Finally, WT^1–166^ had a comparatively reduced CER of 2.14, although this value remained increased as compared to WT^1–433^ (p<0.01, Figure 2B). Together these findings suggest that additional residues between position 20 and 63 promote chromosome localization of cyclin B1, whereas sequences between 110 and 166 tend to antagonize association of these fragments with mitotic chromosomes. Furthermore, since the chromosome association of WT^1–166^ is greater than for WT^1–433^, sequence elements downstream of position 166 also appear to independently antagonize chromosome association of cyclin B1.

We next evaluated the consequences of systematically deleting N–terminal sequences from cyclin B1. As expected based on the previous results, cyclin B1 lacking the first 20 amino acids (WT^21–433^) was strongly excluded from mitotic chromosomes, with a mean CER of 1.35 (Figure 2C, 2D, S1C). Further deletion of twenty-one amino acids (WT^42–433^) led to an even lower degree of chromosome association (mean CER of 1.20; Figure 2C, 2D, S1C). These results are consistent with our data above suggesting that two distinct elements in the N-terminal 41 amino acids of cyclin B1 are important for promoting chromosome localization. Interestingly, the mean CER value for WT^42–433^ (1.20) was lower than for GFP alone (1.37) (p<0.001), consistent with the idea that sequences downstream in cyclin B1 actively promote exclusion of cyclin B1 from chromosomes.

Because the WT^1–166^ and WT^1–110^ fragments exhibited increased association with mitotic chromosomes compared to the full-length WT^1–433^ protein as detailed above, we also assessed the effect of the N-terminal Δ20 and Δ41 deletions in these protein fragments. Compared to WT^1–166^ (mean CER  =  2.14), WT^21–166^ showed reduced chromosome association with a mean CER of 1.66 and WT^42–166^ showed a further reduction in association with a mean CER of 1.37 (Figure 2C, 2D, S1C; p<0.001). A similar pattern was evident for the WT^1–110^ fragment (mean CER = 2.34), where the WT^21–110^ fragment had a mean CER of 1.81 and the WT^42–110^ was further reduced to a mean CER of 1.39 (Figure 2C, 2D, S1C). These effects correlated with our qualitative visual assay, in which WT^21–166^ (mean CER = 1.66) appeared to be excluded from mitotic chromosomes as compared to WT^21–110^ (mean CER = 1.81), which appeared to associate with mitotic chromosomes. However, the signal from the latter protein also appeared dispersed onto the mitotic spindle (Figure 2C and S1C). Taken together, our data suggest sequence elements within cyclin B1 can both positively and negatively regulate its association with mitotic chromosomes.

### Identification of an N–terminal sequence motif in cyclin B1 that is necessary for chromosome localization

**Figure 3:**
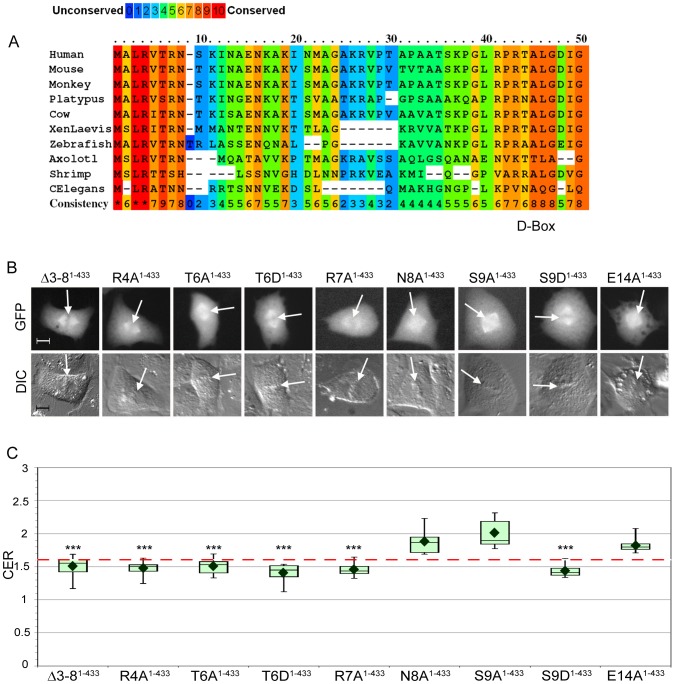
Identification of a novel chromosome localization motif in the N-terminus of cyclin B1. **A**. Alignment of the cyclin B1 protein from a diversity of organisms reveals a region of high conservation within the first 10 amino acids. Degree of conservation for any given position is color-coded from blue (unconserved) to red (highly conserved), and is also indicated with a numerical score in the last row (Consistency). **B**. The Δ3–8 and many single amino acid substitutions in this region specifically disrupt the association of cyclin B1 with mitotic chromosomes. Representative images of mitotic BS-C-1 cells expressing mutated cyclin B1. White arrows indicate position of metaphase plate as seen in the DIC image. Scale bar = 10 µm. **C**. CER box-and-whisker plots for the constructs shown in part B. The red dotted line indicates the threshold of 1.6. N  = 8–10, *** indicates p<0.001 compared to WT^1–433^. Further details including mean CER, standard deviations, and Wilcoxon exact test p-values can be found in Table S2.

To identify specific sequence elements in the N-terminal region of cyclin B1 that could mediate its association with chromosomes, we compared the sequences of the N-terminal 50 amino acids of cyclin B1 from a diversity of organisms and found that amino acids 3–8 (LRVTRN in human) of cyclin B1 are conserved (Figure 3A) in most vertebrates, and some amino acids such as Arg4 and Thr6 are found in lower organisms such as shrimp and nematodes. A homologous sequence is not found in *Drosophila*, plants, or yeast, although the overall similarity between cyclin B in these species and human cyclin B1 is quite low. To determine if this conserved sequence element is necessary for cyclin B1 localization to mitotic chromosomes, we deleted amino acids 3–8 from full-length cyclin B1-GFP and found that localization of Δ3–8^1–433^ to mitotic chromosomes was strongly reduced (mean CER = 1.5; p<0.001 compared to WT^1–433^; Figure 3B, 3C, S4A). Other aspects of the behavior of Δ3–8^1–433^ were indistinguishable from WT^1–433^: in interphase cells, Δ3–8^1–433^ was restricted to the cytoplasm, and upon mitotic entry it translocated into the nucleus and localized properly to the centrosomes and mitotic spindle (Figure S3). Qualitatively, it was degraded at the end of mitosis with normal timing (Figure S3). These experiments indicate that this short, evolutionarily conserved sequence motif within the N–terminus of cyclin B1 is specifically required for the interaction of cyclin B1 with mitotic chromosomes.

To determine whether individual amino acids within this motif play a critical role in mediating the association with mitotic chromosomes, we made a series of single alanine substitutions within the LRVTRN sequence. Preliminary analysis indicated that deletion of the Leu3 residue did not alter localization to mitotic chromosomes (data not shown). In contrast, single alanine mutations of the highly conserved Arg4 (R4A), Thr6 (T6A), or Arg7 (R7A) residues in WT^1–433^ each disrupted association with chromosomes in a manner similar to Δ3–8^1–433^ (Figure 3B, 3C, S4A). In each case, the mutant cyclin B1-GFP was excluded from chromosomes, but still associated with the centrosomes and the mitotic spindle and had a mean CER around 1.5 (Figure 3C), similar to what we observed for deletion of amino acids 3–8. Furthermore, mutation of R4 or R7 to lysine (R4K and R7K), which preserves the positive charge, also caused a loss of association with chromosomes (Figure S4A). In contrast, alanine mutagenesis of amino acids further downstream in the protein-Asn8 (N8A), Glu14 (E14A) and Asn15 (N14A)-did not affect localization to mitotic chromosomes (Figure 3B, 3C, S4A). Thr6 and Ser9 are the only two putative phosphorylation sites within the first 20 amino acids of cyclin B1. As shown above, the T6A mutation disrupted chromosome association of cyclin B1, but the S9A mutation did not, with a mean CER of 2.01 (Figure 3B, 3C, S4A). We also tested phosphomimetic substitutions (aspartic acid and glutamic acid) at the Thr6 and Ser9 positions. Individual mutation of either position (T6D, T6E, S9D, S9E) was sufficient to disrupt chromosome association of cyclin B1 (Figure 3B, 3C, S4A), suggesting that phosphorylation of this sequence has the potential to negatively regulate cyclin B1 interaction with chromosomes. Together these data reveal that single amino acid mutations within the small N-terminal motif of cyclin B1 are capable of specifically disrupting its chromosome localization during mitosis.

**Figure 4:**
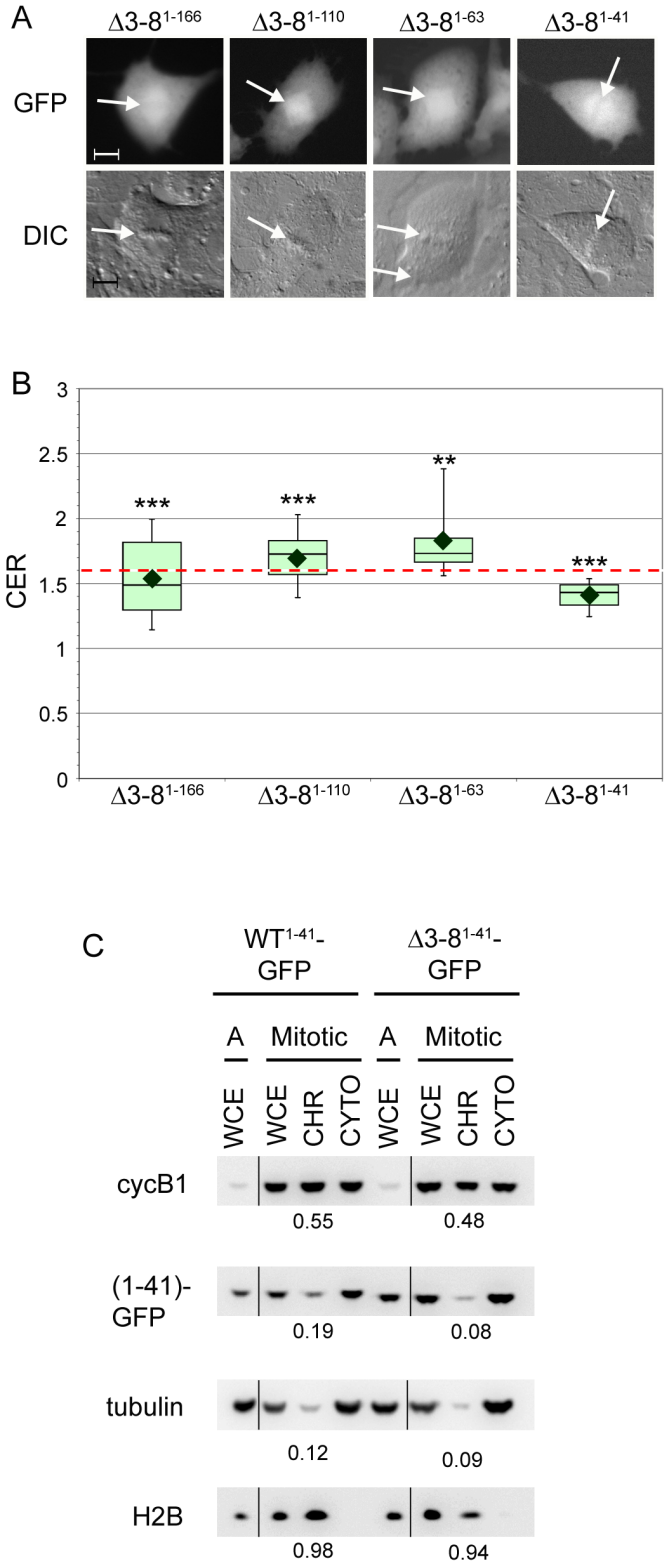
The Δ3–8 mutation causes defects in chromosome localization of N-terminal cyclin B1 fragments. **A**. The Δ3–8 mutation disrupts the chromosome association of cyclin B1 truncations in a context-dependent fashion. Representative images of mitotic BS-C-1 cells expressing mutated cyclin B1. White arrows indicate position of metaphase plate as seen in the DIC image. Scale bar = 10 µm. **B**. CER box-and-whisker plots for the constructs shown in part A. The red dotted line indicates the threshold of 1.6. N  = 8–14, ** indicates p<0.01 and *** indicates p<0.001 compared to WT counterpart. Further details including mean CER, standard deviations, and Wilcoxon exact test p-values can be found in Table S2. **C**. Biochemical fractionation of HeLa cells stably expressing WT^1–41^-GFP or Δ3–8^1–41^-GFP. (1–41)-GFP was detected with a cyclin B1 antibody. (Abbreviations as in Figure 1).

We next introduced the Δ3–8 deletion into the WT^1–166^, WT^1–110^, WT^1–63^, and WT^1–41^ cyclin B1 fragments to determine if it was possible to disrupt their chromosome association. In each case, the Δ3–8 mutation caused a statistically significant decrease in the CER measurement (Figure 3B). Δ3–8^1–166^ and Δ3–8^1–41^ appeared excluded from chromosomes in the qualitative assay (Figure 4A, S4B), with respective mean CERs of 1.54 and 1.42 (Figure 4B). In contrast, Δ3–8^1–63^ and Δ3–8^1–110^ retained some association with chromosomes consistent with greater mean CER values of 1.83 and 1.70, respectively (Figure 4A, 4B, S4B). The appearance of the Δ3–8^1–63^ and Δ3–8^1–110^ signal on the chromosomes was consistently more dispersed than that of the WT^1–63^ and WT^1–110^ counterparts, with a change in distribution from chromosomes to the mitotic spindle (Figure 4A compared to Figure 2A). The appearance of Δ3–8^1–110^-expressing metaphase cells resembled that of WT^21–110^-expressing metaphase cells (compare Figure 4A with 2C), suggesting that the chromosome association in this context is mediated by elements downstream of position 21. Together, these results indicate that the Δ3–8 mutation alters the chromosome localization behavior of all the cyclin B1 fragments, but the degree of the defect depends on the context in which the mutation is examined.

To confirm the importance of this LRVTRN motif in promoting cyclin B1 association with mitotic chromosomes, we used the cell fractionation approach (as in Figure 1) and examined the association of the GFP fusion proteins with mitotic chromosomes. We were unable to generate stable cell lines expressing full-length cyclin B1-GFP, but we successfully generated HeLa cells that stably express the WT^1–41^-GFP or Δ3–8^1–41^-GFP fragment of cyclin B1. Live cell fluorescence imaging demonstrated that the transgenes were well tolerated and the cells underwent normal mitosis (data not shown). Consistent with the results obtained with transfected cells, fluorescence imaging indicated that the WT^1–41^-GFP protein was clearly associated with the mitotic chromosomes, whereas the Δ3–8^1–41^-GFP mutant protein was not (Figure S5). We fractionated the stable cell lines and used quantitative Western blotting to determine the levels of cyclin B1 and the GFP-tagged transgenes in the chromosomal and cytoplasmic fractions. About 20% of total WT^1–41^-GFP was present in the mitotic chromosome fraction (Figure 4C). In contrast, only 8% of mutant Δ3–8^1–41^-GFP was found in the mitotic chromosome fraction, which is similar to the cytoplasmic contamination in this experiment (9% as measured by tubulin; Figure 4C). These findings support the data obtained from live cell imaging of transfected BSC1 cells, and demonstrate that the Δ3–8 mutation is capable of disrupting the mitotic chromosome association of the WT^1–41^ cyclin B1 fragment.

Because WT^1–20^, WT^1–41^, WT^1–110^ and WT^1–166^ associated with mitotic chromosomes to different extents, we next examined the effect of individual point mutations at positions 4–7 in each of these different contexts. Because a large number of constructs were analyzed, we relied on only the qualitative assay, as our previous experiments showed a strong correlation between the qualitative and quantitative assays. In the context of WT^1–20^, amino acid substitutions such as R4A, R4K, T6A, R7A and R7K each disrupted chromosome association, just as we had observed for full-length cyclin B1 (Figure S6A). Furthermore, mutations such as N8A or S9A, which had no effect on the chromosome association of full-length cyclin B1, showed no defect in the localization behavior of WT^1–20^ (Figure S6A). In addition, proteins bearing the phosphomimetic substitutions at Thr6 and Ser9 (T6D, T6E, S9D, S9E) were all found to be excluded from chromosomes (Figure S6A). Thus, all of the single amino acid substitutions tested in the WT^1–20^ cyclin B1 fragment behaved the same way as they did in the context of full-length cyclin B1.

In the context of WT^1–41^, two of the alanine substitutions, R4A and R7A, and the phosphomimetic mutations T6D, T6E, S9D and S9E all disrupted chromosome localization (Figure S6B), as they had in WT^1–433^ and WT^1–20^. Similarly, the N8A and S9A mutations had no effect (Figure S6B) as seen with WT^1–433^ and WT^1–20^. However, unlike what we observed for WT^1–433^ and WT^1–20^, the T6A, R4K and R7K mutations did not strongly disrupt the chromosome localization of WT^1–41^ (Figure S6B). These findings are consistent with the idea that WT^1–41^ contains an additional chromosome binding element that can partially compensate for weaker binding induced by some of these mutations. This trend was further accentuated in the context of WT^1–110^, which associates more strongly with chromosomes. Consistent with our earlier findings that Δ3–8^1–110^– and WT^21–110^-expressing cells retain some chromosome association (Figure 3D, 4A), we found that none of the point mutations in WT^1–110^ caused a disruption in association with chromosomes (Figure S7). However, mutations such as the phosphomimetic substitutions at Thr6 and Ser9 caused WT^1–110^ to have a diffuse signal around the chromosomes, similar to the appearance of the Δ3–8^1–110^- and WT^21–110^-expressing cells (data not shown). Interestingly, in the context of the WT^1–166^ fragment, which shows decreased chromosome association compared to WT^1–110^, most of the mutations again disrupted chromosome association, although some exhibited mixed phenotypes (Figure S6C). Together these findings are fully consistent with the context-dependence of the effects that we observed in characterizing the Δ3–8 mutation in these fragments.

### Mutations in and near the D-box of cyclin B1 disrupt chromosome localization

**Figure 5:**
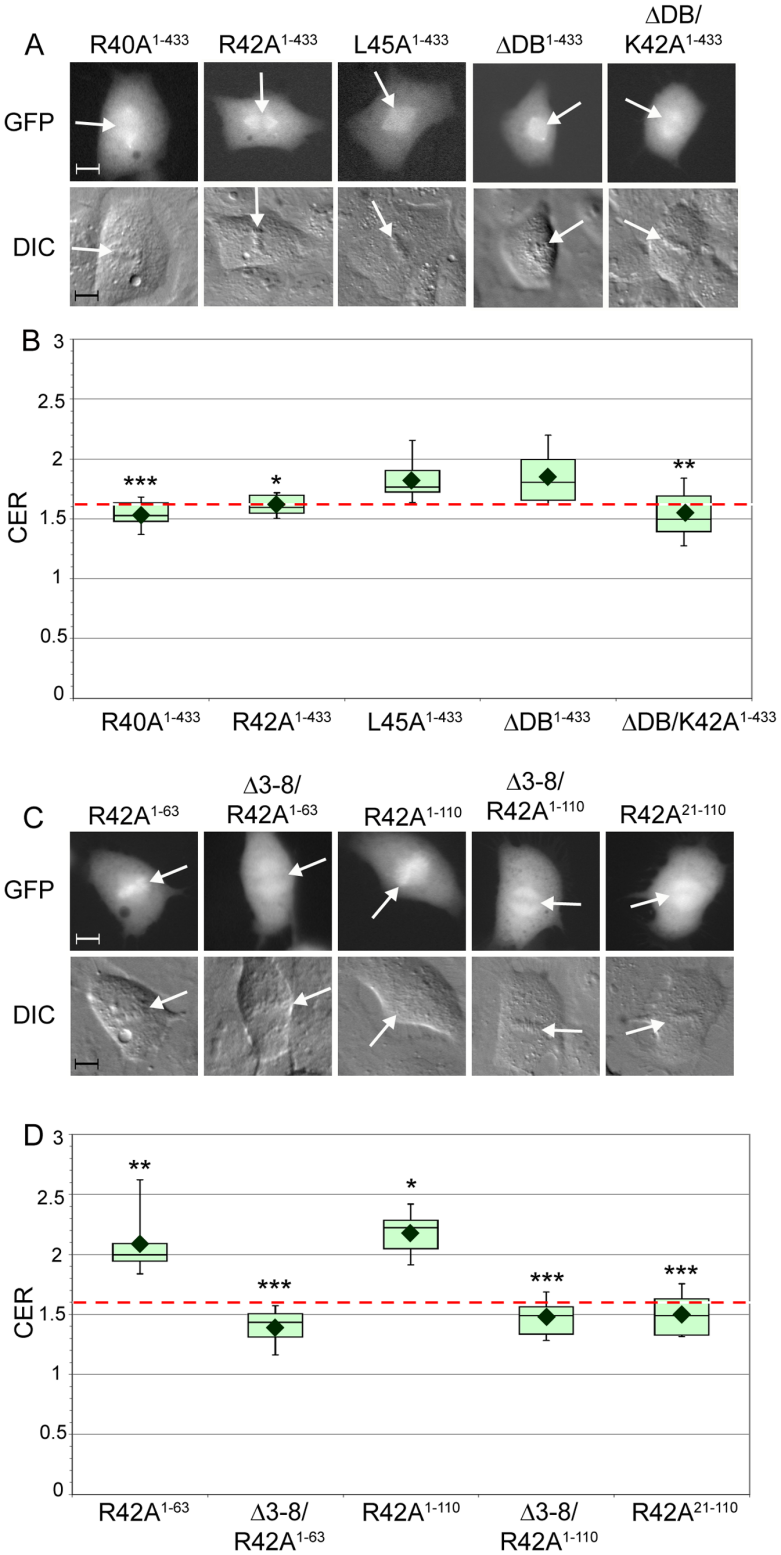
Arginine residues in and near the D-box are required for cyclin B1 localization to mitotic chromosomes. **A**. Representative images of mitotic BS-C-1 cells expressing cyclin B1 containing mutations in and near the D-box. White arrows indicate the position of the metaphase plate as seen in the DIC image. Scale bar = 10 µm. **B**. CER box-and-whisker plots for the constructs shown in part A. The red dotted line indicates the threshold of 1.6. N  = 10–14, * indicates p<0.05, ** indicates p<0.01 and *** indicated p<0.001 compared to WT^1–433^. Further details including mean CER, standard deviations, and Wilcoxon exact test p-values can be found in Table S2. **C**. Representative images of mitotic BS-C-1 cells expressing cyclin B1 fragments bearing R42A or Δ3–8/R42A double mutations. White arrows indicate the position of the metaphase plate as seen in the DIC image. Scale bar = 10 µm. **D**. CER box-and-whisker plots for the constructs shown in part C. The red dotted line indicates the threshold of 1.6. N = 9–13, * indicates p<0.05, ** indicated p<0.01 and ***indicates p<0.001 compared to WT counterpart. Further details including mean CER, standard deviations, and Wilcoxon exact test p-values can be found in Table S2.

Cyclin B1's destruction box (D-box) sequence is essential for its recognition by the APC/C and subsequent ubiquitin-dependent degradation at the end of mitosis [31]. This conserved RXXLXXXXN sequence is found at amino acids 42–50 in human cyclin B1. Because cyclin B1 fragments including this region (WT^1–63^ and WT^1–110^) showed increased association with mitotic chromosomes compared to WT^1–41^, which lacks the D-box, we tested the effects of D-box mutations on cyclin B1 localization. Previous studies examining the localization of cyclin B1-GFP bearing D-box mutations produced contradictory results. Clute and Pines [11] showed that the single point mutant R42A cyclin B1-GFP had disrupted chromosome association, whereas we found that deletion of the entire 9-amino acid D-box sequence did not perturb chromosome association [9]. To address this conflict, we examined three non-degradable D-box mutants in the context of full length cyclin B1: the single point mutants R42A (R42A^1–433^) and L45A (L45A^1–433^) and a 9-amino acid deletion of the entire D-box (ΔDB^1–433^). As previously shown [9], [11], cells expressing cyclin B1 bearing D-box mutations arrest in mitosis due to the inability to degrade cyclin B1 and inactivate CDK1. Consistent with the previously reported findings [11], proteins bearing the D-box mutations exhibited distinct chromosome association behavior. R42A^1–433^ cyclin B1 was excluded from mitotic chromosomes in a manner resembling the Δ3–8^1–433^ mutation and had a mean CER of 1.61 (Figure 5A, 5B, S8A). In contrast, L45A^1–433^ and ΔDB^1–433^ retained association with mitotic chromosomes, with mean CERs of 1.82 and 1.85, respectively (Figure 5A, 5B, S8A). In the qualitative assay, there appeared to be a partial defect in the localization behavior of ΔDB^1–433^ because there was a significant increase in the percentage of cells that lacked mitotic chromosome association (2% of WT^1–433^-expressing cells and 21% of ΔDB^1–433^-expressing cells; Fisher's exact p-value  = 1.3×10^−11^; Figure S8A). We noticed that in the ΔDB^1–433^ sequence, a lysine (K51 in the wild-type sequence) now resides at position 42. To test whether the positive charge at position 42 plays a role in conferring chromosome localization, we mutagenized ΔDB^1–433^ so that the lysine at position 42 was changed to alanine (ΔDB/K42A^1–433^). ΔDB/K42A^1–433^ had a mean CER of 1.54 and did not associate with mitotic chromosomes in the qualitative assay, in a manner that resembled R42A^1–433^ (Figure 5A, 5B). Taken together, these data indicated that positively charged amino acids in the D-box play a role in mediating chromosome association during mitosis.

We next wondered whether positively charged amino acids are required specifically in the context of the D-box, or whether neighboring positively charged amino acids also contribute to chromosome association. Furthermore, our previous analysis had suggested that amino acids between 21 and 41 could promote association with mitotic chromosomes. We therefore analyzed localization of the R40A^1–433^ mutation. Cells expressing R40A^1–433^ underwent normal mitosis and R40A^1–433^-GFP was degraded at the metaphase to anaphase transition (data not shown), indicating that this residue is not required for mitotic destruction of cyclin B1. However, R40A^1–433^ was excluded from mitotic chromosomes in a manner similar to R42A^1–433^ with a mean CER of 1.53 (Figure 5A, 5B, S8A). These data indicate that positively charged residues neighboring the D-box are also involved in the mitotic chromosome localization of cyclin B1, and that chromosome localization of cyclin B1 is unlikely to be mediated by its interaction with the APC/C.

As described earlier, the chromosome localization of some cyclin B1 N-terminal fragments (such as WT^1–63^ or WT^1–110^) was not fully disrupted by mutation of the conserved LRVTRN sequence element in the N-terminus, suggesting that other sequences in these N-terminal fragments mediate chromosome localization. To assess whether the destruction box is important for the chromosome localization of these fragments, we introduced the R42A point mutation into WT^1–63^ and WT^1–110^. R42A^1–63^ had a mean CER of 2.09 and R42A^1–110^ had a mean CER of 2.18. In both cases, these values were significantly decreased as compare to their WT counterparts (Figure 5D), although the magnitude of the change was not as dramatic as for Δ3–8^1–63^ and Δ3–8^1–110^. In the qualitative assay, we observed that R42A^1–63^ and R42A^1–110^ retained chromosome association (Figure 5C, S8B), as we had found for the same fragments bearing the Δ3–8 mutation (Figure 4A, S4B). As with the Δ3–8^1–63^ and Δ3–8^1–110^, there was a qualitative change in the appearance of the chromosomal signal as compared to wild type, where there was increased signal on the mitotic spindle (Figure 5C). Since neither the Δ3–8 mutation nor the R42A mutation was independently capable of fully disrupting the chromosome localization of WT^1–63^ or WT^1–110^, we tested whether these mutations could act together to disrupt chromosome localization. Indeed, constructs encoding Δ3–8/R42A^1–63^ and Δ3–8/R42A^1–110^ were strongly excluded from mitotic chromosomes, with mean CER values of 1.39 and 1.48, respectively (Figure 5C, 5D, S8B). We also introduced the R42A mutation into WT^21–110^, and found that R42A^21–110^ was excluded from mitotic chromosomes, with a mean CER of 1.50 Figure 5C, 5D, S8B. Taken together, these data indicate that both the N-terminal LRVTRN motif (amino acids 3–8) and basic residues in and around the destruction box play important roles in promoting the chromosome association of cyclin B1.

## Discussion

**Figure 6:**
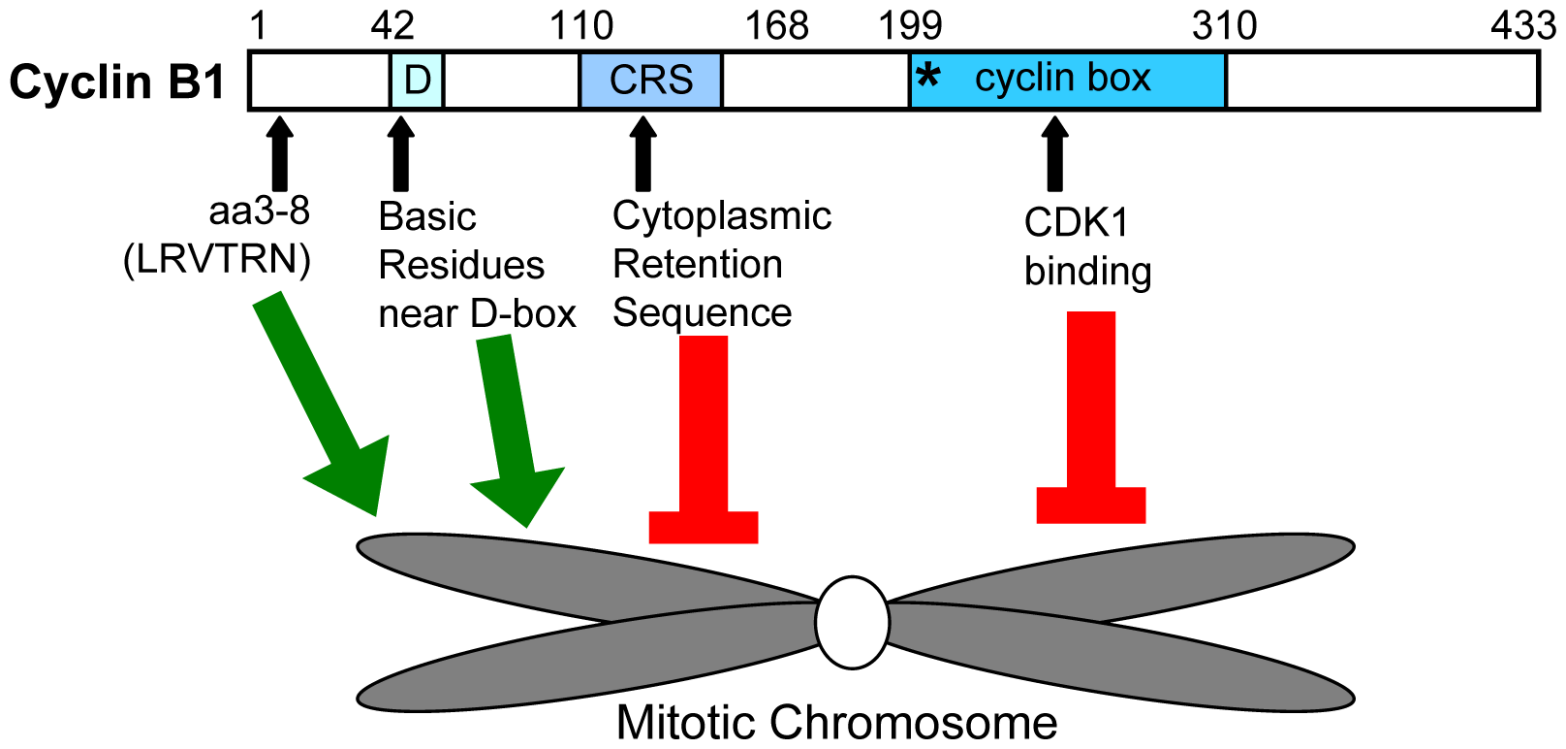
Model summarizing key findings. A schematic representation of the Cyclin B1 protein. The numbers along the top indicate amino acid positions. D = D-box sequence, CRS = cytoplasmic retention sequence, * = MRAIL motif. This paper identifies amino acids 3–8 (LRVTRN) and arginine residues near the D-box as sequence motifs that promote cyclin B1 localization to chromosomes, whereas downstream regions, including the MRAIL motif, the CRS and the cyclin box may antagonize chromosome association.

Previous studies of cyclin B1 localization by our lab and others [9], [11] established that cyclin B1-CDK1 associates with chromosomes during prometaphase and metaphase. Using a biochemical fractionation approach in mitotic HeLa cells, we found that between 30–40% of endogenous cyclin B1 and CDK1 are associated with the chromosome-enriched fraction. Because many proteins associated with mitotic chromosomes are CDK1 substrates, the localization of cyclin B1-CDK1 to chromosomes is likely to play an important functional role in mitotic progression. Therefore, we sought to identify the sequence elements in cyclin B1 that are required for its recruitment to chromosomes. Together our data suggest a model in which cyclin B1 is like a rope in a molecular tug of war (Figure 6). Basic residues in the N-terminal domain facilitate cyclin B1 association with chromosomes, whereas sequence elements downstream of position 110 tend to antagonize chromosome association. This finely tuned balance may help make cyclin B1 localization susceptible to regulation, as our data suggest that phosphorylation in or near the N-terminal motif would be sufficient antagonize chromosome localization. In contrast, phosphorylation at positions downstream of 110 may help promote chromosome association, as discussed below.

We have found that the first 20 amino acids of cyclin B1 serves as a minimal region sufficient for targeting to mitotic chromosomes, but that association is enhanced by other regions in the N-terminal domain, including arginine residues in and near the destruction box. Within the N-terminal 20 amino acids, we identified a small, evolutionarily conserved motif (aa3–8, LRVTRN) that is necessary for localization of full-length cyclin B1 to mitotic chromosomes. Analysis of point mutations indicates that the two arginines and threonine are important within this sequence, and that phosphorylation in this region might negatively regulate association with mitotic chromatin.

Our work identifies the importance of several arginine residues (Arg4, Arg7, Arg40, Arg42) in the N-terminal domain of cyclin B1 in mediating chromosome association, but we do not yet understand how these residues promote chromosome association. Short arginine motifs can insert into the minor groove of DNA, where the guanidinium side chains of arginine associate with the negatively charged phosphate backbone though both electrostatic interactions and hydrogen bonding [33], [34]. For example, homeodomain proteins are defined by a canonical Helix-Turn-Helix DNA-binding domain, but also have short, unstructured arginine-rich N-terminal arms that insert into the minor groove of DNA [35]. One such protein, Engrailed, has an Arg-Pro-Arg motif in the N-terminal arm [33], [36] that is identical to amino acids 40–42 of cyclin B1. Therefore, it is possible that arginine residues within the N-terminal unstructured region of cyclin B1 interact with the minor groove of DNA in a similar manner as these homeobox proteins. Overall, the N-terminal 110 amino acids of cyclin B1 is highly positively charged, with an isoelectric point of 9.9, which is consistent with the possibility of an electrostatic interaction with DNA. The basic N-terminal region of cyclin B1 is reminiscent of that of RCC1, whose N-terminal region has been shown to interact directly with DNA [16], [37]. In the case of RCC1, N-terminal methylation, which enhances the basicity of the sequence, also appears to be important for DNA binding [29]. Whether cyclin B1 is modified in a similar manner remains to be determined. However, stable interaction of RCC1 with chromatin also involves interaction of the downstream folded domain of the protein with core histones [38].

Alternatively, the N-terminal motif and additional arginine residues could mediate a specific protein-protein interaction that tethers cyclin B1 to chromatin. In support of this idea, mutation of threonine 6 to alanine disrupted chromosome association. Furthermore, even mutations that retained the positive charge at positions 4 and 7 of cyclin B1 (mutation of arginine to lysine) were found to be sufficient to disrupt cyclin B1 association with chromosomes. Another possibility is that the arginine-rich motifs in cyclin B1 could mediate interactions with phosphorylated proteins on mitotic chromosomes. Arginine-phosphate interactions have been proposed to be a common mechanism underlying protein-protein interactions, and the stability of such interactions can be very strong when measured with peptides [39]. Many CDK1 substrates are known to associate with mitotic chromosomes. If one of these phosphorylated proteins recruits the cyclin B1-CDK1 complex to chromosomes, this mechanism could yield a positive feedback loop in which CDK1-dependent phosphorylation of chromosome proteins promotes further association of the cyclin B1-CDK1 complex with chromosomes.

The identity of proteins that might recruit cyclin B1 to chromosomes remains unclear. Separase is known to associate with chromosomes [23], [24], and to bind stably to the cyclin B1/CDK1 complex after it is phosphorylated by CDK1 [20], [21]. This interaction is thought to be especially important for inhibiting separase that is not bound by securin. However, a cyclin mutant lacking the first 90 amino acids is capable of binding and inhibiting separase, suggesting that the cyclin B1 N-terminal domain does not mediate this interaction [21]. Other known mitotic chromosome proteins such as condensins or topoisomerase II could also serve to recruit cyclin B1, but a role for the cyclin B1 N-terminal domain in such interactions remains to be determined. In an effort to identify potential chromosome receptor proteins for cyclin B1, we attempted to purify proteins that interact specifically with WT^1–166^-GFP compared to Δ3–8^1–166^-GFP, but these experiments failed to identify proteins enriched for specific binding to the wild-type protein.

Because arginine at position 42 in the destruction box is required for both APC-dependent proteolysis of cyclin B1 and its localization to chromosomes, it is possible that cyclin B1 localization to chromosomes could be mediated by the APC. Some components of the APC, such as APC3, have been reported to interact with mitotic chromosomes [40], [41], [42], [43]. However, Cdc20, an important component of the D-box receptor, does not appear to be enriched on chromosomes during mitosis, except at the kinetochore where its binding is highly dynamic [44]. Furthermore, we identified a destruction box mutation (L45A) that stabilized cyclin B1 in mitosis without affecting its localization to chromosomes. We thus favor the model that recruitment of cyclin B1 to chromosomes is not mediated by destruction box-dependent interactions with the APC. Instead, Arg42 may play distinct roles in recruiting cyclin B1 to chromosomes and in binding to the APC. This model suggests that binding of cyclin B1 to chromosomes and the APC could be mutually exclusive.

At the other end of the tug-of-war, our data suggest that sequence elements downstream of position 110 in cyclin B1 negatively modulate chromosome association, as progressive deletion of C-terminal sequences tends to enhance chromosome association. For example, WT^1–166^ exhibits increased chromosome association as compared to the full length protein (WT^1–433^), and WT^1–110^ exhibits increased association with mitotic chromosome as compared to WT^1–166^. The same pattern occurs for fragments that lack the first 20 or 40 amino acids. There are several functional domains in the C-terminal region of the protein that may have roles in antagonizing the chromosome association behavior of the N-terminal region: the MRAIL motif (residues 201–205), the cyclin box (residues 199–310), and the cytoplasmic retention sequence (CRS, residues 110–160). The MRAIL motif is important for cyclin B1 localization to centrosomes [9]. It is possible that recruitment of cyclin B1 to centrosomes may antagonize its ability to localize to chromosomes. The cyclin box is required for binding to CDK1, and the CDK subunit may have affinity for other substrates or structures in the cell that would also tend to antagonize chromosome association. The CRS plays an important role in maintaining cyclin B1 in the cytoplasm during interphase [8]. The CRS appears to be functional in the WT^1–166^-GFP protein because it is excluded from the nucleus during interphase whereas WT^1–110^-GFP is constitutively nuclear (Figure S2). This region of cyclin B1 that has been shown to bind the nuclear exportin CRM1 [45], and binding of CRM1 has been shown to be negatively regulated by phosphorylation of cyclin B1 in the CRS [46]. Interestingly, mutation of five phosphorylation sites of cyclin B1 (Ser 116, 126, 128, 133,147) to alanine abolishes the association of cyclin B1 with mitotic chromosomes [47]. One model consistent with these data is that phosphorylation of the CRS in cyclin B1 abolishes binding of a protein-possibly CRM1-that antagonizes its association with mitotic chromatin. Understanding how cyclin B1 binding to CRM1 might antagonize chromosome association is an interesting future question. In this model, phosphorylation of cyclin B1 in this region would promote both nuclear translocation and also mitotic chromosome association, and may be important for the switch-like nature of mitotic entry [47].

## Supporting Information

Figure S1 **Qualitative analysis assessing mitotic chromosome localization of cyclin B1 fragments in BS-C-1 cells. A**. Representative images of BS-C-1 cells expressing WT^1–433^-GFP that were scored as “cannot call”. **B**. Graphical representation of qualitative analysis showing the distribution of chromosome localization behavior for all mitotic cells expressing cyclin B1 fragments shown in Figure 2A. **C**. Graphical representation of qualitative analysis showing the distribution of chromosome localization behavior for all mitotic cells expressing cyclin B1 fragments shown in Figure 2C. For statistical analysis of these data, see Table S1.
(TIF)

Figure S2 **Summary and interphase localization of Cyclin B1 fragments used in this paper.** **A**. Schematic representation of the cyclin B1 protein. The relative arrangement of the key protein domains (D = D-box; CRS = cytoplasmic retention sequence; * = MRAIL motif, cyclin box = CDK1 binding domain) is indicated and the localization properties of the cyclin B1 fragments examined in this paper are noted. **B**. Localization of transfected cyclin B1-GFP fragments in BS-C-1 cells during interphase. WT^1–41^-GFP, WT^1–63^-GFP, and WT^-110^-GFP lack the CRS sequences and have prominent nuclear accumulation in interphase cells. WT^1–166^-GFP and WT^1–433^-GFP include the CRS sequences and exhibit localization only in the cytoplasm of interphase cells. Scale bar = 10 µm.
(TIF)

Figure S3 **Localization of WT^1–433^ and Δ3–8^1–433^ during mitotic progression.** Time lapse images taken at 10 minute intervals of BS-C-1 cells expressing WT^1–433^-GFP (**A**) and Δ3–8^1–433^-GFP (**B**). Accumulation of GFP in the nucleus and at centrosomes is evident in the first frame of mitosis (0′) for both WT^1–433^-GFP and Δ3–8^1–433^-GFP. A. WT^1–433^ is present on mitotic chromosomes throughout metaphase until the cyclin B1 is degraded. B. Δ3–8^1–433^-GFP is specifically excluded from mitotic chromosomes, but all other localization and degradation behavior appears normal. CER measurements were performed on the first frame of metaphase. Scale bar = 10 µm.
(TIF)

Figure S4 **Qualitative analysis of Δ3–8 and N-terminal single amino acid mutations of cyclin B1.** **A**. Graphical representation showing the distribution of chromosome localization behavior for all mitotic BS-C-1 cells expressing all mutant full-length cyclin B1 constructs utilized in this study. Single amino acid mutations in WT^1–433^ disrupt mitotic chromosome localization in all cases except N8A^1–433^, S9A^1–433^, E14A^1–433^ and N15A^1–433^. Even the conservative lysine substitution in positions R4 and R7 cause a disruption in mitotic chromosome association. Representative images and quantitative analysis for Δ3–8^1–433^, R4A^1–433^, T6A^1–433^, T6D^1–433^, R7A^1–433^, N8A^1–433^, S9A^1–433^, S9D^1–433^, E14A^1–433^ can be found in Figure 3B and 3C, respectively. **B**. Graphical representation showing the distribution of chromosome localization behavior for all mitotic BS–C-1 cells expressing Δ3–8 cyclin B1 fragments. Δ3–8^1–166^ and Δ3–8^1–41^ are excluded from mitotic chromosomes, whereas Δ3–8^1–110^ and Δ3–8^1–63^ retain chromosome association. Note that the chromosome localization of Δ3–8^1–110^ and Δ3–8^1–63^ has a blurred appearance (Figure 4A) and the CER values are significantly reduced compared to their wild–type counterparts (Figure 4B). Representative images and quantitative analysis for these constructs can be found in Figure 4A and 4B, respectively. For statistical analysis of these data, see Table S1.
(TIF)

Figure S5 **Mitotic chromosome localization of HeLa cells stably expressing WT^1–41^-GFP and Δ3–8^1–41^-GFP.** Localization of cyclin B1 derivatives expressed from stable transgenes is consistent with that seen in transfected BS-C-1 cells (Figures 2A and 4A). Stable cell lines were imaged by time lapse and selected metaphase cells are shown. White arrows indicate location of the metaphase plate. Scale bar = 10 µm.
(TIF)

Figure S6 **Mutagenesis of individual conserved amino acids in cyclin B1 fragments can disrupt mitotic chromosome localization. A**. Graphical representation showing the distribution of chromosome localization behavior for mitotic BS-C-1 cells expressing mutant cyclin B1^1–20^ constructs. Single amino acid mutations in WT^1–20^ disrupt mitotic chromosome localization in all cases except N8A^1–20^ and S9A^1–20^. For reference, WT^1–20^ exhibited positive chromosome association in 74% of expressing mitotic cells (Figure S1B). **B**. Graphical representation showing the distribution of chromosome localization behavior for mitotic BS-C-1 cells expressing mutant cyclin B1^1–41^ constructs. Single amino acid mutations in WT^1–41^ disrupt mitotic chromosome localization in the cases of R4A, R7A, and the phosphomimetic substitutions T6D, T6E, S9D, S9E. For reference, WT^1–41^ exhibited positive chromosome association in 90% of expressing mitotic cells (Figure S1B). **C**. Graphical representation showing the distribution of chromosome localization behavior for mitotic BS-C-1 cells expressing mutant cyclin B1^1–166^ constructs. Single amino acid mutations in WT^1–166^ cause a range of localization behaviors. R4K, T6A, R7K, N8A, and S9A mutations have normal association with mitotic chromosomes. T6D and R7A are strongly excluded from mitotic chromosomes. R4A, T6E, S9D, and S9E mutations have partial exclusion phenotypes. For reference, WT^1–166^ exhibited positive chromosome association in 94% of expressing mitotic cells (Figure S1B). For statistical analysis of these data, see Table S1.
(TIF)

Figure S7 **N-terminal single amino acid substitutions do not fully disrupt chromosome localization of WT^1–110^.** Graphical representation showing the distribution of chromosome localization behavior for mitotic BS-C-1 cells expressing mutant cyclin B1^1–110^ constructs. All mutants exhibit mitotic chromosome association. For statistical analysis of these data, see Table S1.
(TIF)

Figure S8 **Qualitative analysis of full-length and truncated cyclin B1 bearing mutations in and proximal to the D-box. A**. Graphical representation showing the distribution of chromosome localization for BS-C-1 mitotic cells expressing cyclin B1 mutants shown in Figure 5A. R40A^1–433^ and R42A^1–433^ are excluded from mitotic chromosomes, whereas L45A^1–433^ and ΔDB^1–433^ largely retain localization to mitotic chromosomes. Further mutation of ΔDB^1–433^ to ΔDB/K42A^1–433^ causes a delocalization from mitotic chromosomes. For statistical analysis of these data, see Table S1. **B**. Graphical representation showing the distribution of chromosome localization for BS-C-1 mitotic cells expressing cyclin B1 fragments bearing R42A or Δ3–8/R42A double mutations shown in Figure 5C. R42A^1–63^ and R42A^1–110^ retain chromosome localization, but Δ3–8/R42A^1–63^ and Δ3–8/R42A^1–110^ are excluded from mitotic chromosomes. R42A^21–110^ is also excluded from mitotic chromosomes. For statistical analysis of these data, see Table S1.
(TIF)

Table S1 **Statistical analysis of qualitative live cell imaging data.**.
(PDF)

Table S2 **Wilcoxon exact test p-values for quantitative analysis.**
(PDF)

## References

[1] Morgan DO (2007) The Cell Cycle: Principles of Control; Lawrence E, editor. Corby: Oxford University Press. 297 p.

[2] Lindqvist A, Rodriguez-Bravo V, Medema RH (2009) The decision to enter mitosis: feedback and redundancy in the mitotic entry network. J Cell Biol 185: 193–202. 19364923 10.1083/jcb.200812045 PMC2700378

[3] Hartl P, Gottesfeld J, Forbes DJ (1993) Mitotic repression of transcription in vitro. J Cell Biol 120: 613–624. 8381119 10.1083/jcb.120.3.613 PMC2119533

[4] Pyronnet S, Dostie J, Sonenberg N (2001) Suppression of cap-dependent translation in mitosis. Genes Dev 15: 2083–2093. 11511540 10.1101/gad.889201 PMC312759

[5] Nigg EA (2001) Mitotic kinases as regulators of cell division and its checkpoints. Nat Rev Mol Cell Biol 2: 21–32. 11413462 10.1038/35048096

[6] Elia AE, Cantley LC, Yaffe MB (2003) Proteomic screen finds pSer/pThr-binding domain localizing Plk1 to mitotic substrates. Science 299: 1228–1231. 12595692 10.1126/science.1079079

[7] Pines J, Hunter T (1991) Human cyclins A and B1 are differentially located in the cell and undergo cell cycle-dependent nuclear transport. J Cell Biol 115: 1–17. 1717476 10.1083/jcb.115.1.1 PMC2289910

[8] Pines J, Hunter T (1994) The differential localization of human cyclins A and B is due to a cytoplasmic retention signal in cyclin B. Embo J. 13: 3772–3781. 10.1002/j.1460-2075.1994.tb06688.x PMC395290 8070405

[9] Bentley AM, Normand G, Hoyt J, King RW (2007) Distinct sequence elements of cyclin B1 promote localization to chromatin, centrosomes, and kinetochores during mitosis. Mol Biol Cell 18: 4847–4858. 17881737 10.1091/mbc.E06-06-0539 PMC2096604

[10] Chen Q, Zhang X, Jiang Q, Clarke PR, Zhang C (2008) Cyclin B1 is localized to unattached kinetochores and contributes to efficient microtubule attachment and proper chromosome alignment during mitosis. Cell Res 18: 268–280. 18195732 10.1038/cr.2008.11

[11] Clute P, Pines J (1999) Temporal and spatial control of cyclin B1 destruction in metaphase. Nat Cell Biol 1: 82–87. 10559878 10.1038/10049

[12] Hutchins JR, Moore WJ, Hood FE, Wilson JS, Andrews PD, et al (2004) Phosphorylation regulates the dynamic interaction of RCC1 with chromosomes during mitosis. Curr Biol 14: 1099–1104. 15203004 10.1016/j.cub.2004.05.021

[13] Li HY, Zheng Y (2004) Phosphorylation of RCC1 in mitosis is essential for producing a high RanGTP concentration on chromosomes and for spindle assembly in mammalian cells. Genes Dev 18: 512–527. 15014043 10.1101/gad.1177304 PMC374234

[14] Dasso M (2001) Running on Ran: nuclear transport and the mitotic spindle. Cell 104: 321–324. 11239388 10.1016/s0092-8674(01)00218-5

[15] Kalab P, Heald R (2008) The RanGTP gradient – a GPS for the mitotic spindle. J Cell Sci 121: 1577–1586. 18469014 10.1242/jcs.005959 PMC7185306

[16] Moore W, Zhang C, Clarke PR (2002) Targeting of RCC1 to chromosomes is required for proper mitotic spindle assembly in human cells. Curr Biol 12: 1442–1447. 12194828 10.1016/s0960-9822(02)01076-x

[17] Abe S, Nagasaka K, Hirayama Y, Kozuka-Hata H, Oyama M, et al (2011) The initial phase of chromosome condensation requires Cdk1-mediated phosphorylation of the CAP-D3 subunit of condensin II. Genes Dev 25: 863–874. 21498573 10.1101/gad.2016411 PMC3078710

[18] Kimura K, Hirano M, Kobayashi R, Hirano T (1998) Phosphorylation and activation of 13S condensin by Cdc2 in vitro. Science 282: 487–490. 9774278 10.1126/science.282.5388.487

[19] Liu W, Tanasa B, Tyurina OV, Zhou TY, Gassmann R, et al (2010) PHF8 mediates histone H4 lysine 20 demethylation events involved in cell cycle progression. Nature 466: 508–512. 20622854 10.1038/nature09272 PMC3059551

[20] Boos D, Kuffer C, Lenobel R, Korner R, Stemmann O (2008) Phosphorylation-dependent binding of cyclin B1 to a Cdc6-like domain of human separase. J Biol Chem 283: 816–823. 17974570 10.1074/jbc.M706748200

[21] Gorr IH, Boos D, Stemmann O (2005) Mutual inhibition of separase and Cdk1 by two-step complex formation. Mol Cell 19: 135–141. 15989971 10.1016/j.molcel.2005.05.022

[22] Holland AJ, Taylor SS (2006) Cyclin-B1-mediated inhibition of excess separase is required for timely chromosome disjunction. J Cell Sci 119: 3325–3336. 16868023 10.1242/jcs.03083

[23] Sun Y, Kucej M, Fan HY, Yu H, Sun QY, et al (2009) Separase is recruited to mitotic chromosomes to dissolve sister chromatid cohesion in a DNA-dependent manner. Cell 137: 123–132. 19345191 10.1016/j.cell.2009.01.040 PMC2673135

[24] Yuan K, Li N, Huo Y, Yan F, Yang Y, et al (2009) Recruitment of separase to mitotic chromosomes is regulated by Aurora B. Cell Cycle. 8: 1433–1443. 10.4161/cc.8.9.8331 19342897

[25] Escargueil AE, Plisov SY, Skladanowski A, Borgne A, Meijer L, et al (2001) Recruitment of cdc2 kinase by DNA topoisomerase II is coupled to chromatin remodeling. Faseb J 15: 2288–2290. 11511510 10.1096/fj.00-0726fje

[26] Rattner JB, Lew J, Wang JH (1990) p34cdc2 kinase is localized to distinct domains within the mitotic apparatus. Cell Motil Cytoskeleton 17: 227–235. 2268875 10.1002/cm.970170309

[27] Stals H, Bauwens S, Traas J, Van Montagu M, Engler G, et al (1997) Plant CDC2 is not only targeted to the pre-prophase band, but also co-localizes with the spindle, phragmoplast, and chromosomes. FEBS Lett 418: 229–234. 9428718 10.1016/s0014-5793(97)01368-9

[28] Bailly E, Pines J, Hunter T, Bornens M (1992) Cytoplasmic accumulation of cyclin B1 in human cells: association with a detergent-resistant compartment and with the centrosome. J Cell Sci 101 (Pt 3): 529–545. 10.1242/jcs.101.3.529 1387877

[29] Chen T, Muratore TL, Schaner-Tooley CE, Shabanowitz J, Hunt DF, et al (2007) N-terminal alpha-methylation of RCC1 is necessary for stable chromatin association and normal mitosis. Nat Cell Biol 9: 596–603. 17435751 10.1038/ncb1572 PMC4624279

[30] Jackman M, Lindon C, Nigg EA, Pines J (2003) Active cyclin B1-Cdk1 first appears on centrosomes in prophase. Nat Cell Biol 5: 143–148. 12524548 10.1038/ncb918

[31] Glotzer M, Murray AW, Kirschner MW (1991) Cyclin is degraded by the ubiquitin pathway. Nature 349: 132–138. 1846030 10.1038/349132a0

[32] Nugent JH, Alfa CE, Young T, Hyams JS (1991) Conserved structural motifs in cyclins identified by sequence analysis. J Cell Sci 99 (Pt 3): 669–674. 10.1242/jcs.99.3.669 1834684

[33] Rohs R, West SM, Sosinsky A, Liu P, Mann RS, et al (2009) The role of DNA shape in protein-DNA recognition. Nature 461: 1248–1253. 19865164 10.1038/nature08473 PMC2793086

[34] West SM, Rohs R, Mann RS, Honig B (2010) Electrostatic interactions between arginines and the minor groove in the nucleosome. J Biomol Struct Dyn 27: 861–866. 20232938 10.1080/07391102.2010.10508587 PMC2946858

[35] Mann RS, Lelli KM, Joshi R (2009) Hox specificity unique roles for cofactors and collaborators. Curr Top Dev Biol 88: 63–101. 19651302 10.1016/S0070-2153(09)88003-4 PMC2810641

[36] Kissinger CR, Liu BS, Martin-Blanco E, Kornberg TB, Pabo CO (1990) Crystal structure of an engrailed homeodomain-DNA complex at 2.8 A resolution: a framework for understanding homeodomain-DNA interactions. Cell 63: 579–590. 1977522 10.1016/0092-8674(90)90453-l

[37] Seino H, Hisamoto N, Uzawa S, Sekiguchi T, Nishimoto T (1992) DNA-binding domain of RCC1 protein is not essential for coupling mitosis with DNA replication. J Cell Sci 102 (Pt 3): 393–400. 10.1242/jcs.102.3.393 1506422

[38] Nemergut ME, Mizzen CA, Stukenberg T, Allis CD, Macara IG (2001) Chromatin docking and exchange activity enhancement of RCC1 by histones H2A and H2B. Science 292: 1540–1543. 11375490 10.1126/science.292.5521.1540

[39] Woods AS, Ferre S (2005) Amazing stability of the arginine-phosphate electrostatic interaction. J Proteome Res 4: 1397–1402. 16083292 10.1021/pr050077s PMC2945258

[40] Huang JY, Morley G, Li D, Whitaker M (2007) Cdk1 phosphorylation sites on Cdc27 are required for correct chromosomal localisation and APC/C function in syncytial Drosophila embryos. J Cell Sci 120: 1990–1997. 17519285 10.1242/jcs.006833 PMC2082081

[41] Topper LM, Campbell MS, Tugendreich S, Daum JR, Burke DJ, et al (2002) The dephosphorylated form of the anaphase-promoting complex protein Cdc27/Apc3 concentrates on kinetochores and chromosome arms in mitosis. Cell Cycle 1: 282–292. 12429948

[42] Huang JY, Raff JW (2002) The dynamic localisation of the Drosophila APC/C: evidence for the existence of multiple complexes that perform distinct functions and are differentially localised. J Cell Sci 115: 2847–2856. 12082146 10.1242/jcs.115.14.2847

[43] Jorgensen PM, Brundell E, Starborg M, Hoog C (1998) A subunit of the anaphase-promoting complex is a centromere-associated protein in mammalian cells. Mol Cell Biol 18: 468–476. 9418894 10.1128/mcb.18.1.468 PMC121516

[44] Kallio MJ, Beardmore VA, Weinstein J, Gorbsky GJ (2002) Rapid microtubule-independent dynamics of Cdc20 at kinetochores and centrosomes in mammalian cells. J Cell Biol 158: 841–847. 12196507 10.1083/jcb.200201135 PMC2173153

[45] Yang J, Bardes ES, Moore JD, Brennan J, Powers MA, et al (1998) Control of cyclin B1 localization through regulated binding of the nuclear export factor CRM1. Genes Dev 12: 2131–2143. 9679058 10.1101/gad.12.14.2131 PMC317017

[46] Yang J, Song H, Walsh S, Bardes ES, Kornbluth S (2001) Combinatorial control of cyclin B1 nuclear trafficking through phosphorylation at multiple sites. J Biol Chem 276: 3604–3609. 11060306 10.1074/jbc.M008151200

[47] Santos SD, Wollman R, Meyer T, Ferrell JE Jr (2012) Spatial positive feedback at the onset of mitosis. Cell 149: 1500–1513. 22726437 10.1016/j.cell.2012.05.028 PMC3395376

