# Supplementary figures and images for: Determinants of Human Cyclin B1 Association with Mitotic Chromosomes
Source: PLoS One. 2013 Mar 11;8(3):e59169. doi: 10.1371/journal.pone.0059169 (PMC3594322; doi:10.1371/journal.pone.0059169)

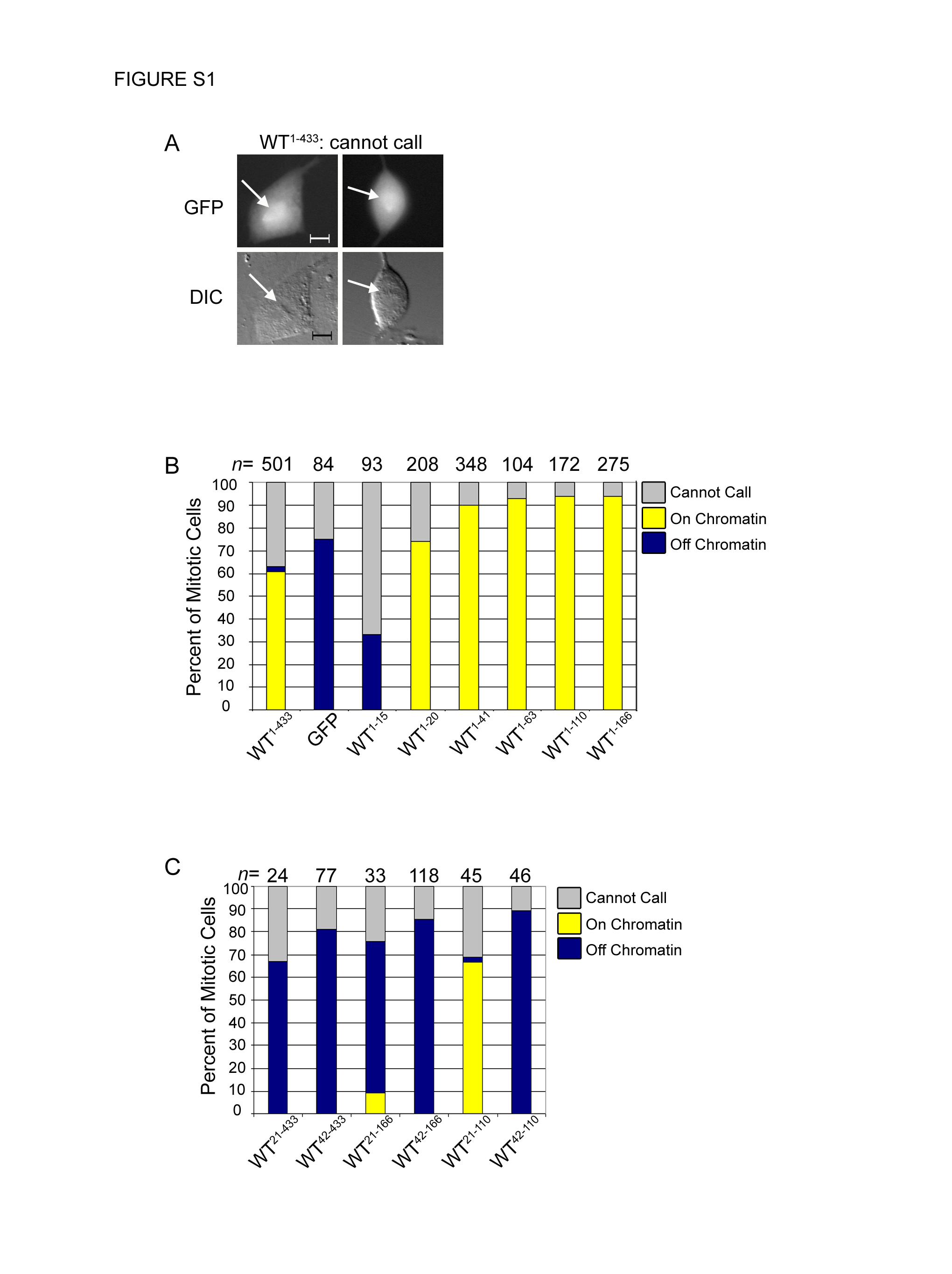

Supplement: Figure S1 — Qualitative analysis assessing mitotic chromosome localization of cyclin B1 fragments in BS-C-1 cells. A. Representative images of BS-C-1 cells expressing WT1–433-GFP that were scored as “cannot call”. B. Graphical representation of qualitative analysis showing the distribution of chromosome localization behavior for all mitotic cells expressing cyclin B1 fragments shown in Figure 2A. C. Graphical representation of qualitative analysis showing the distribution of chromosome localization behavior for all mitotic cells expressing cyclin B1 fragments shown in Figure 2C. For statistical analysis of these data, see Table S1. (TIF) [file pone.0059169.s001.tif]

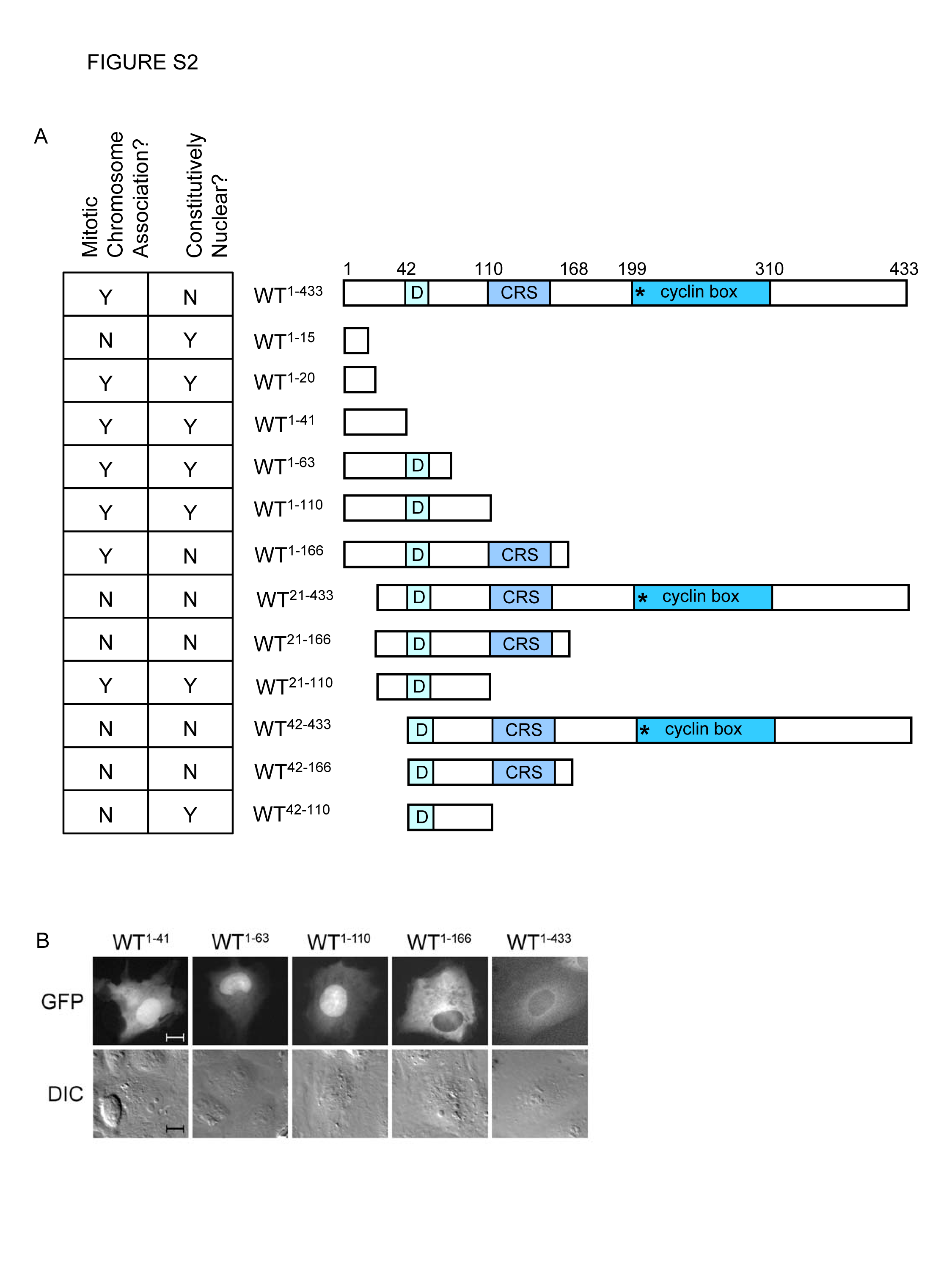

Supplement: Figure S2 — Summary and interphase localization of Cyclin B1 fragments used in this paper. A. Schematic representation of the cyclin B1 protein. The relative arrangement of the key protein domains (D = D-box; CRS = cytoplasmic retention sequence; * = MRAIL motif, cyclin box = CDK1 binding domain) is indicated and the localization properties of the cyclin B1 fragments examined in this paper are noted. B. Localization of transfected cyclin B1-GFP fragments in BS-C-1 cells during interphase. WT1–41-GFP, WT1–63-GFP, and WT-110-GFP lack the CRS sequences and have prominent nuclear accumulation in interphase cells. WT1–166-GFP and WT1–433-GFP include the CRS sequences and exhibit localization only in the cytoplasm of interphase cells. Scale bar = 10 µm. (TIF) [file pone.0059169.s002.tif]

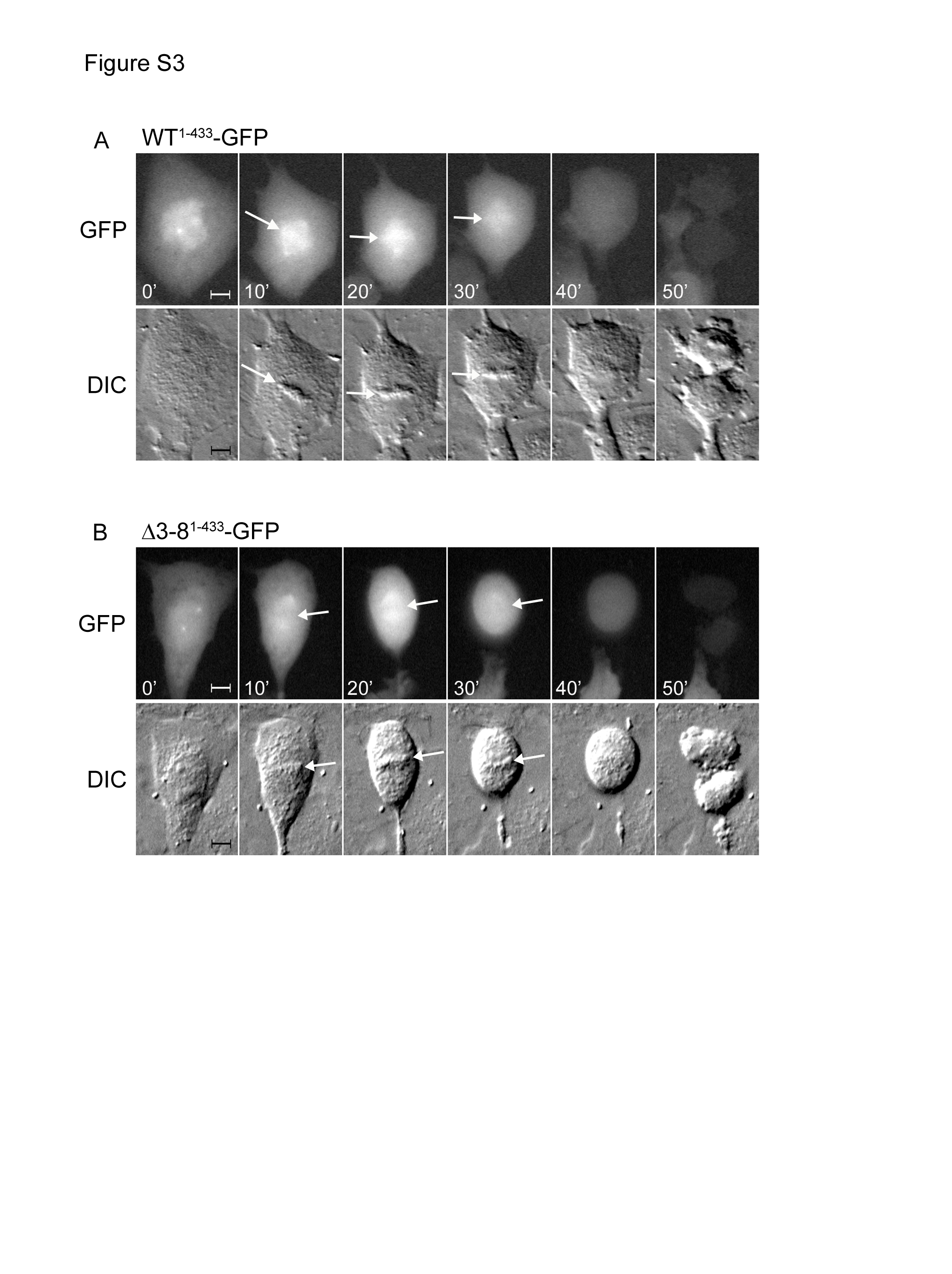

Supplement: Figure S3 — Localization of WT1–433 and Δ3–81–433 during mitotic progression. Time lapse images taken at 10 minute intervals of BS-C-1 cells expressing WT1–433-GFP (A) and Δ3–81–433-GFP (B). Accumulation of GFP in the nucleus and at centrosomes is evident in the first frame of mitosis (0′) for both WT1–433-GFP and Δ3–81–433-GFP. A. WT1–433 is present on mitotic chromosomes throughout metaphase until the cyclin B1 is degraded. B. Δ3–81–433-GFP is specifically excluded from mitotic chromosomes, but all other localization and degradation behavior appears normal. CER measurements were performed on the first frame of metaphase. Scale bar = 10 µm. (TIF) [file pone.0059169.s003.tif]

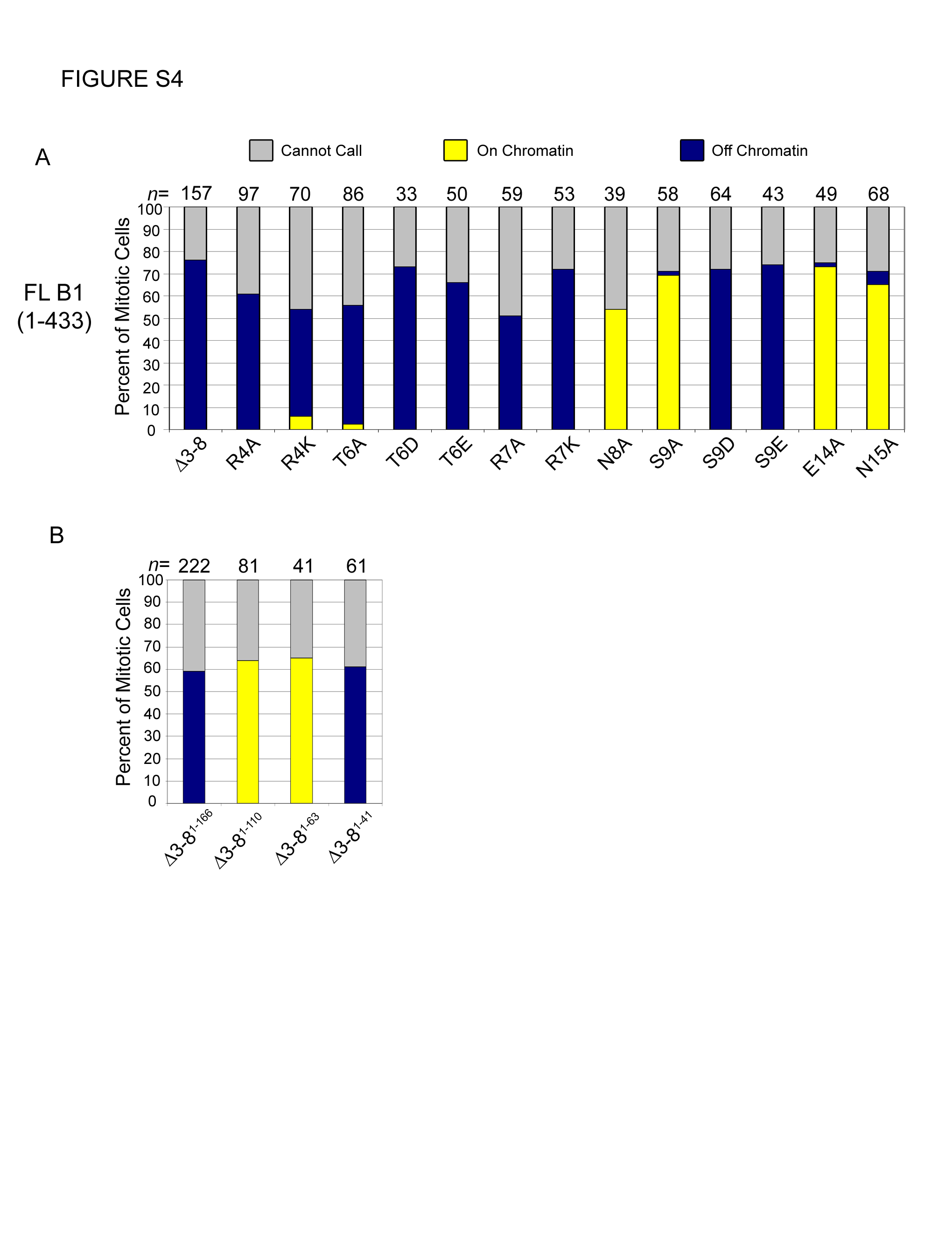

Supplement: Figure S4 — Qualitative analysis of Δ3–8 and N-terminal single amino acid mutations of cyclin B1. A. Graphical representation showing the distribution of chromosome localization behavior for all mitotic BS-C-1 cells expressing all mutant full-length cyclin B1 constructs utilized in this study. Single amino acid mutations in WT1–433 disrupt mitotic chromosome localization in all cases except N8A1–433, S9A1–433, E14A1–433 and N15A1–433. Even the conservative lysine substitution in positions R4 and R7 cause a disruption in mitotic chromosome association. Representative images and quantitative analysis for Δ3–81–433, R4A1–433, T6A1–433, T6D1–433, R7A1–433, N8A1–433, S9A1–433, S9D1–433, E14A1–433 can be found in Figure 3B and 3C, respectively. B. Graphical representation showing the distribution of chromosome localization behavior for all mitotic BS–C-1 cells expressing Δ3–8 cyclin B1 fragments. Δ3–81–166 and Δ3–81–41 are excluded from mitotic chromosomes, whereas Δ3–81–110 and Δ3–81–63 retain chromosome association. Note that the chromosome localization of Δ3–81–110 and Δ3–81–63 has a blurred appearance (Figure 4A) and the CER values are significantly reduced compared to their wild–type counterparts (Figure 4B). Representative images and quantitative analysis for these constructs can be found in Figure 4A and 4B, respectively. For statistical analysis of these data, see Table S1. (TIF) [file pone.0059169.s004.tif]

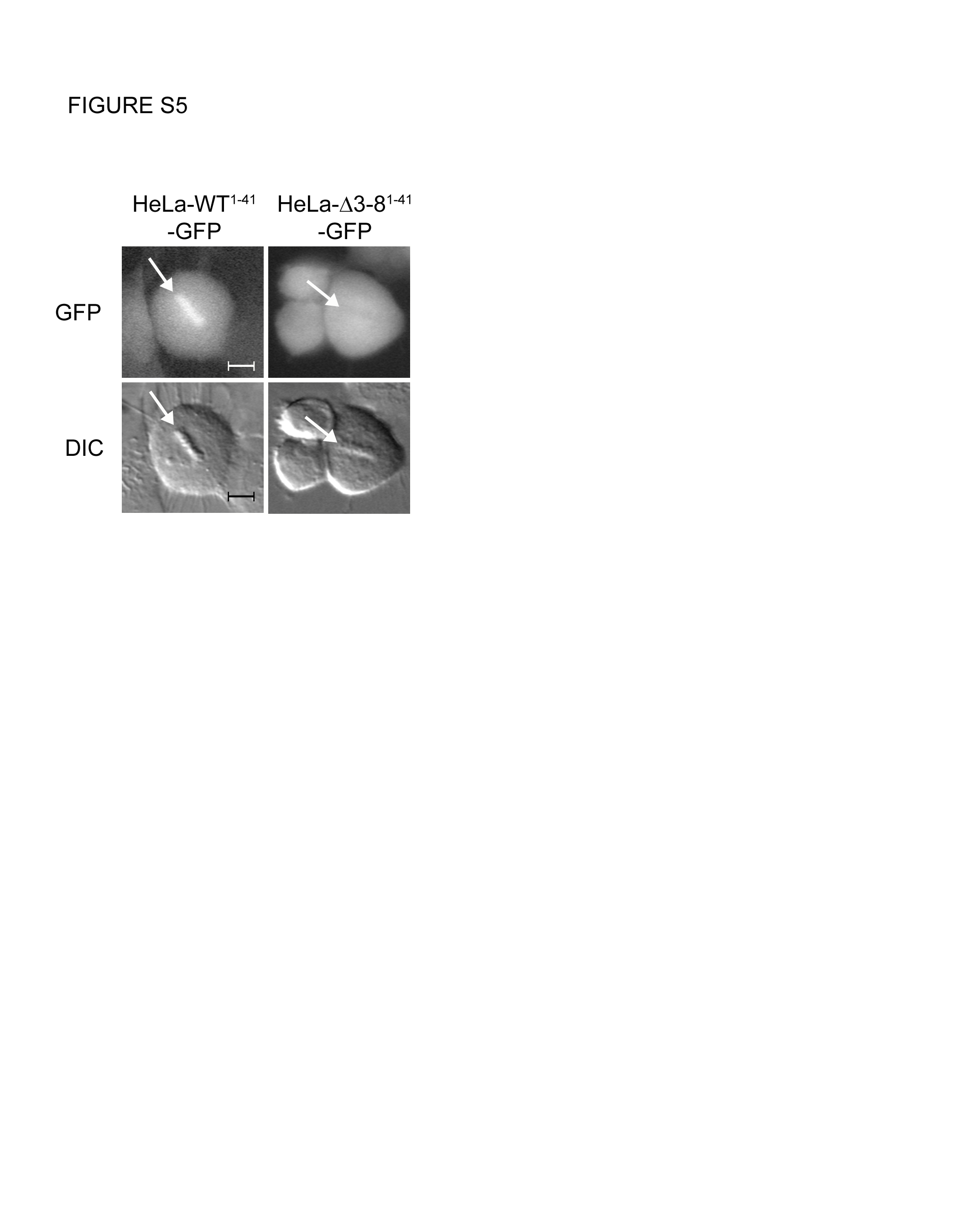

Supplement: Figure S5 — Mitotic chromosome localization of HeLa cells stably expressing WT1–41-GFP and Δ3–81–41-GFP. Localization of cyclin B1 derivatives expressed from stable transgenes is consistent with that seen in transfected BS-C-1 cells (Figures 2A and 4A). Stable cell lines were imaged by time lapse and selected metaphase cells are shown. White arrows indicate location of the metaphase plate. Scale bar = 10 µm. (TIF) [file pone.0059169.s005.tif]

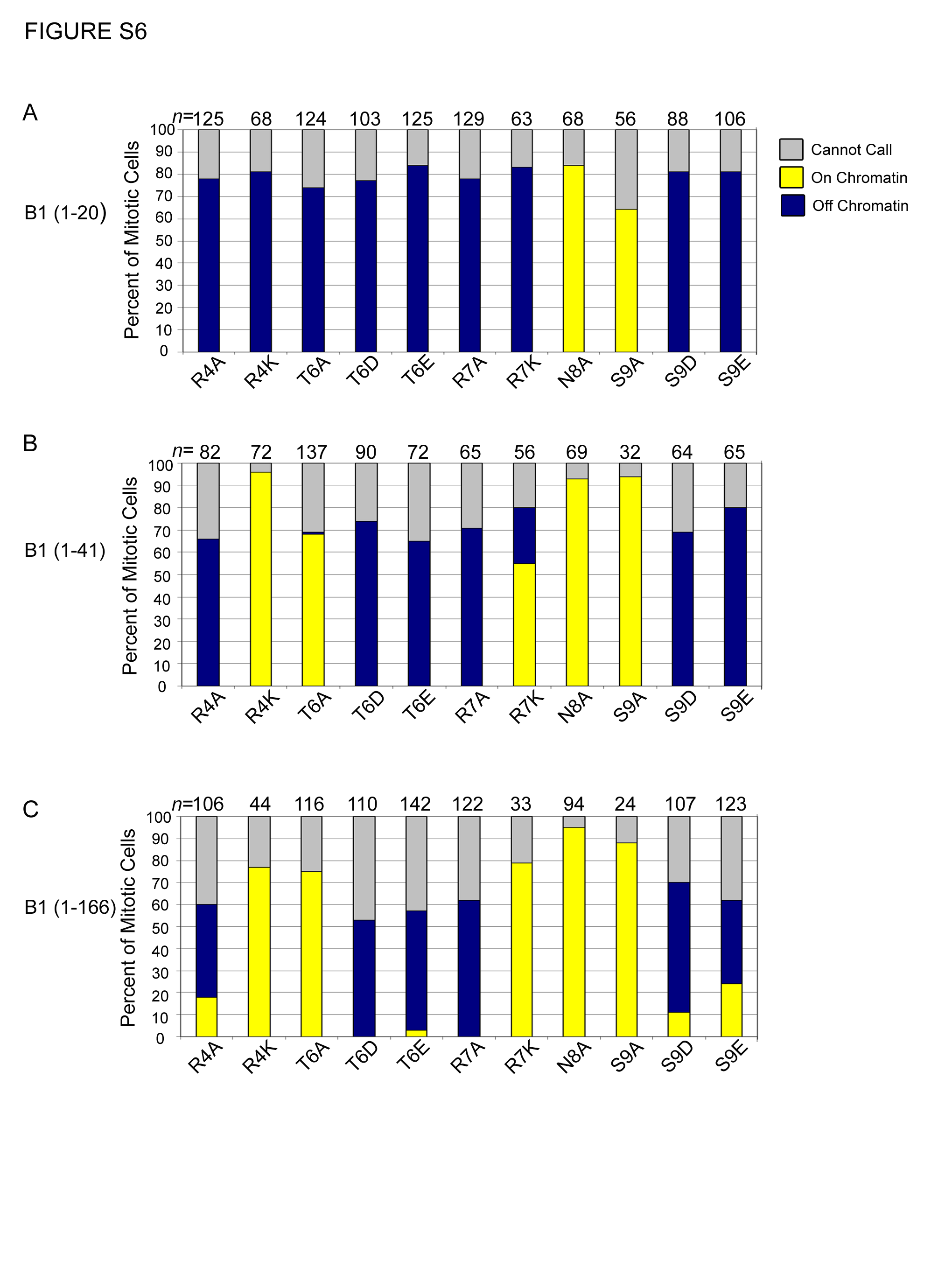

Supplement: Figure S6 — Mutagenesis of individual conserved amino acids in cyclin B1 fragments can disrupt mitotic chromosome localization. A. Graphical representation showing the distribution of chromosome localization behavior for mitotic BS-C-1 cells expressing mutant cyclin B11–20 constructs. Single amino acid mutations in WT1–20 disrupt mitotic chromosome localization in all cases except N8A1–20 and S9A1–20. For reference, WT1–20 exhibited positive chromosome association in 74% of expressing mitotic cells (Figure S1B). B. Graphical representation showing the distribution of chromosome localization behavior for mitotic BS-C-1 cells expressing mutant cyclin B11–41 constructs. Single amino acid mutations in WT1–41 disrupt mitotic chromosome localization in the cases of R4A, R7A, and the phosphomimetic substitutions T6D, T6E, S9D, S9E. For reference, WT1–41 exhibited positive chromosome association in 90% of expressing mitotic cells (Figure S1B). C. Graphical representation showing the distribution of chromosome localization behavior for mitotic BS-C-1 cells expressing mutant cyclin B11–166 constructs. Single amino acid mutations in WT1–166 cause a range of localization behaviors. R4K, T6A, R7K, N8A, and S9A mutations have normal association with mitotic chromosomes. T6D and R7A are strongly excluded from mitotic chromosomes. R4A, T6E, S9D, and S9E mutations have partial exclusion phenotypes. For reference, WT1–166 exhibited positive chromosome association in 94% of expressing mitotic cells (Figure S1B). For statistical analysis of these data, see Table S1. (TIF) [file pone.0059169.s006.tif]

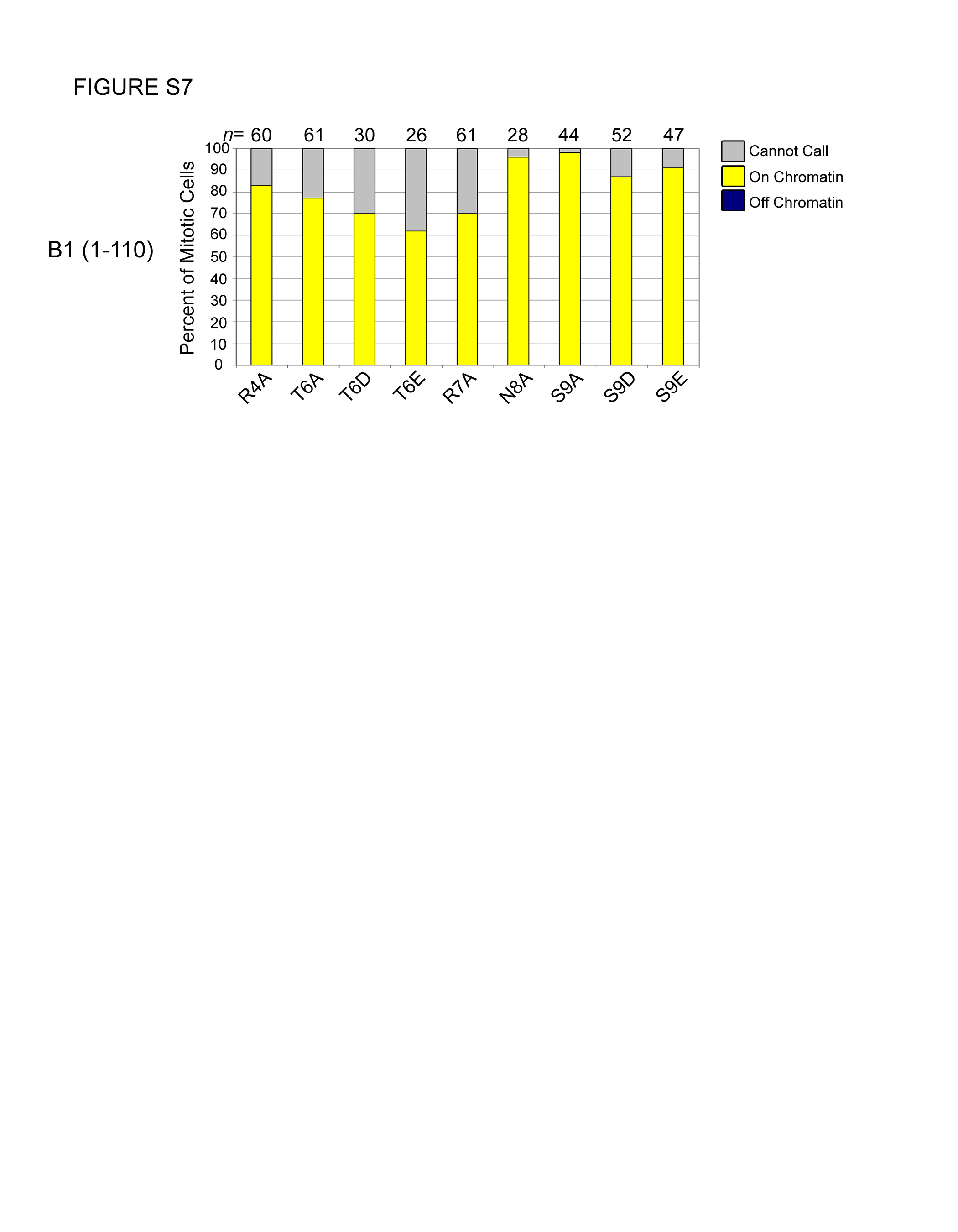

Supplement: Figure S7 — N-terminal single amino acid substitutions do not fully disrupt chromosome localization of WT1–110. Graphical representation showing the distribution of chromosome localization behavior for mitotic BS-C-1 cells expressing mutant cyclin B11–110 constructs. All mutants exhibit mitotic chromosome association. For statistical analysis of these data, see Table S1. (TIF) [file pone.0059169.s007.tif]

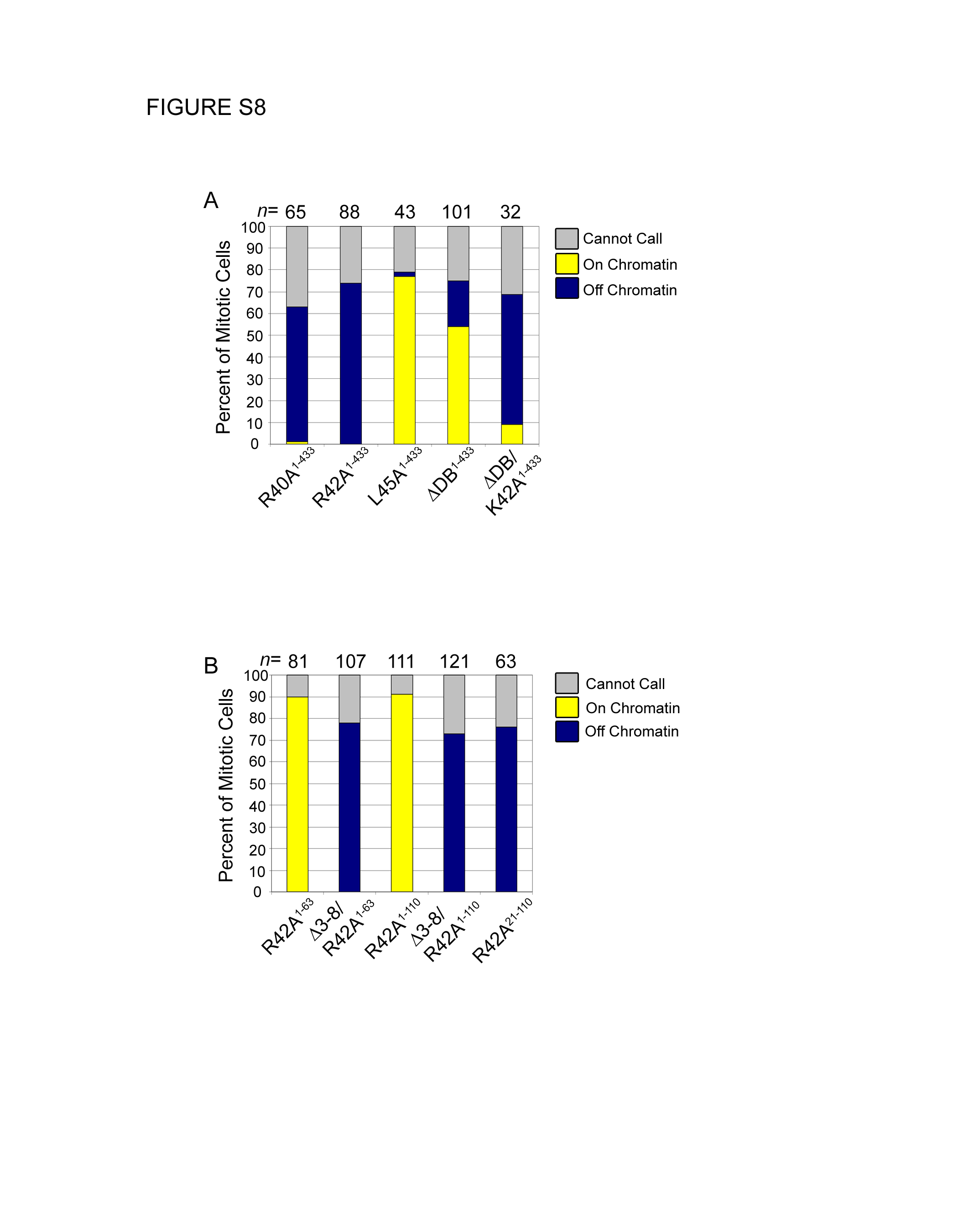

Supplement: Figure S8 — Qualitative analysis of full-length and truncated cyclin B1 bearing mutations in and proximal to the D-box. A. Graphical representation showing the distribution of chromosome localization for BS-C-1 mitotic cells expressing cyclin B1 mutants shown in Figure 5A. R40A1–433 and R42A1–433 are excluded from mitotic chromosomes, whereas L45A1–433 and ΔDB1–433 largely retain localization to mitotic chromosomes. Further mutation of ΔDB1–433 to ΔDB/K42A1–433 causes a delocalization from mitotic chromosomes. For statistical analysis of these data, see Table S1. B. Graphical representation showing the distribution of chromosome localization for BS-C-1 mitotic cells expressing cyclin B1 fragments bearing R42A or Δ3–8/R42A double mutations shown in Figure 5C. R42A1–63 and R42A1–110 retain chromosome localization, but Δ3–8/R42A1–63 and Δ3–8/R42A1–110 are excluded from mitotic chromosomes. R42A21–110 is also excluded from mitotic chromosomes. For statistical analysis of these data, see Table S1. (TIF) [file pone.0059169.s008.tif]
