# Supplementary material for: Determinants of Human Cyclin B1 Association with Mitotic Chromosomes
Source: PLoS One. 2013 Mar 11;8(3):e59169. doi: 10.1371/journal.pone.0059169 (PMC3594322; doi:10.1371/journal.pone.0059169)
Supplement: Table S1 — Statistical analysis of qualitative live cell imaging data.. (PDF) [file pone.0059169.s009.pdf]

**Table S1. Statistical analysis of qualitative live cell imaging data**. Fisher's exact p-values for qualitative analysis. Mitotic BS-C-1 cells expressing GFP-tagged constructs were visually scored for the presence or absence of chromosome association. P-values comparing the frequency of cells exhibiting chromosome association were determined using a Fisher's exact test as described in the Methods. Abbreviations: N1(ON CHR) = number of cells expressing construct 1 with positive chromosome association; N1(OFF CHR + CC) = number of cells expressing construct 1 that had exclusion from mitotic chromosome or were "cannot call". N2(ON CHR) = number of cells expressing construct 2 with positive chromosome association; N2(OFF CHR + CC) = number of cells expressing construct 2 that had exclusion from mitotic chromosome or were "cannot call". The "OFF CHR" and "Cannot Call" were combined in order to have a 2-variable analysis that compared the frequency of cells that exhibited chromosome association.

| Figure | Construct 1 | N1 (ON CHR) | N1 (OFF CHR +CC) | Construct 2 | N2 (ON CHR) | N2 (OFF CHR +CC) | Fisher's Exact Test p-value |
|---|---|---|---|---|---|---|---|
| Figure S1B | $WT^{1-433}$ | 308 | 193 | GFP | 0 | 84 | $2.15 \times 10^{-31}$ |
| | | | | $WT^{1-15}$ | 0 | 93 | $2.79 \times 10^{-34}$ |
| | | | | $WT^{1-20}$ | 154 | 54 | $1.35 \times 10^{-3}$ |
| | | | | $WT^{1-41}$ | 314 | 34 | $4.01 \times 10^{-22}$ |
| | | | | $WT^{1-63}$ | 99 | 5 | $1.32 \times 10^{-13}$ |
| | | | | $WT^{1-110}$ | 162 | 10 | $9.42 \times 10^{-19}$ |
| | | | | $WT^{1-166}$ | 258 | 17 | $1.49 \times 10^{-25}$ |
| Figure S1C | $WT^{1-433}$ | 308 | 193 | $WT^{21-433}$ | 0 | 24 | $2.82 \times 10^{-10}$ |
| | | | | $WT^{42-433}$ | 0 | 77 | $4.19 \times 10^{-29}$ |
| | $WT^{1-110}$ | 162 | 10 | $WT^{21-110}$ | 56 | 18 | $9.33 \times 10^{-5}$ |
| | | | | $WT^{42-110}$ | 0 | 46 | $8.82 \times 10^{-38}$ |
| | $WT^{1-166}$ | 258 | 17 | $WT^{21-166}$ | 3 | 30 | $3.13 \times 10^{-26}$ |
| | | | | $WT^{42-166}$ | 0 | 118 | $1.85 \times 10^{-82}$ |
| Figure S4A | $WT^{1-433}$ | 308 | 193 | $\Delta 3\text{-}8^{1-433}$ | 0 | 157 | $4.31 \times 10^{-53}$ |
| | | | | $R4A^{1-433}$ | 0 | 97 | $1.53 \times 10^{-35}$ |
| | | | | $R4K^{1-433}$ | 4 | 66 | $2.50 \times 10^{-20}$ |
| | | | | $T6A^{1-433}$ | 2 | 84 | $2.22 \times 10^{-28}$ |
| | | | | $T6D^{1-433}$ | 0 | 33 | $1.12 \times 10^{-13}$ |
| | | | | $T6E^{1-433}$ | 0 | 50 | $7.30 \times 10^{-20}$ |

**Table S1.** Con't.

| Figure | Construct 1 | N1 (ON CHR) | N1 (OFF CHR +CC) | Construct 2 | N2 (ON CHR) | N2 (OFF CHR +CC) | Fisher's Exact Test p-value |
|---|---|---|---|---|---|---|---|
| Figure S4A (con't) | $WT^{1-433}$ | 308 | 193 | $R7A^{1-433}$ | 0 | 59 | $5.10 \times 10^{-23}$ |
| | | | | $R7K^{1-433}$ | 0 | 53 | $6.35 \times 10^{-21}$ |
| | | | | $N8A^{1-433}$ | 21 | 18 | 0.4 |
| | | | | $S9A^{1-433}$ | 40 | 18 | 0.32 |
| | | | | $S9D^{1-433}$ | 0 | 64 | $9.72 \times 10^{-25}$ |
| | | | | $S9E^{1-433}$ | 0 | 43 | $2.36 \times 10^{-17}$ |
| | | | | $E14A^{1-433}$ | 36 | 13 | 0.12 |
| | | | | $N15A^{1-433}$ | 44 | 24 | 0.69 |
| Figure S1B vs. Figure S4B | $WT^{1-166}$ | 258 | 17 | $\Delta3\text{-}8^{1-166}$ | 0 | 222 | $4.90 \times 10^{-122}$ |
| | $WT^{1-110}$ | 162 | 10 | $\Delta3\text{-}8^{1-110}$ | 59 | 33 | $3.69 \times 10^{-9}$ |
| | $WT^{1-63}$ | 99 | 5 | $\Delta3\text{-}8^{1-63}$ | 48 | 26 | $1.58 \times 10^{-7}$ |
| | $WT^{1-41}$ | 314 | 34 | $\Delta3\text{-}8^{1-41}$ | 0 | 61 | $1.89 \times 10^{-48}$ |
| Figure S6A | $WT^{1-20}$ | 154 | 54 | $R4A^{1-20}$ | 0 | 125 | $6.19 \times 10^{-49}$ |
| | | | | $R4K^{1-20}$ | 0 | 68 | $3.70 \times 10^{-31}$ |
| | | | | $T6A^{1-20}$ | 0 | 124 | $2.95 \times 10^{-48}$ |
| | | | | $T6D^{1-20}$ | 0 | 103 | $1.89 \times 10^{-42}$ |
| | | | | $T6E^{1-20}$ | 0 | 125 | $1.23 \times 10^{-48}$ |
| | | | | $R7A^{1-20}$ | 0 | 129 | $9.79 \times 10^{-50}$ |
| | | | | $R7K^{1-20}$ | 0 | 63 | $2.30 \times 10^{-29}$ |
| | | | | $N8A^{1-20}$ | 57 | 11 | 0.14 |
| | | | | $S9A^{1-20}$ | 36 | 20 | 0.18 |
| | | | | $S9D^{1-20}$ | 0 | 88 | $7.30 \times 10^{-38}$ |
| | | | | $S9E^{1-20}$ | 0 | 106 | $3.39 \times 10^{-43}$ |

**Table S1.** Con't.

| Figure | Construct 1 | N1 (ON CHR) | N1 (OFF CHR +CC) | Construct 2 | N2 (ON CHR) | N2 (OFF CHR +CC) | Fisher's Exact Test p-value |
|---|---|---|---|---|---|---|---|
| Figure S6B | $WT^{1-41}$ | 314 | 34 | $R4A^{1-41}$ | 0 | 82 | $5.07 \times 10^{-61}$ |
| | | | | $R4K^{1-41}$ | 69 | 3 | 0.17 |
| | | | | $T6A^{1-41}$ | 93 | 44 | $1.25 \times 10^{-8}$ |
| | | | | $T6D^{1-41}$ | 0 | 90 | $1.77 \times 10^{-65}$ |
| | | | | $T6E^{1-41}$ | 0 | 72 | $3.33 \times 10^{-55}$ |
| | | | | $R7A^{1-41}$ | 0 | 65 | $5.94 \times 10^{-51}$ |
| | | | | $R7K^{1-41}$ | 31 | 25 | $2.12 \times 10^{-9}$ |
| | | | | $N8A^{1-41}$ | 64 | 5 | 0.65 |
| | | | | $S9A^{1-41}$ | 30 | 2 | 0.75 |
| | | | | $S9D^{1-41}$ | 0 | 64 | $2.48 \times 10^{-50}$ |
| | | | | $S9E^{1-41}$ | 0 | 65 | $5.94 \times 10^{-51}$ |
| Figure S6C | $WT^{1-166}$ | 258 | 17 | $R4A^{1-166}$ | 19 | 87 | $5.01 \times 10^{-49}$ |
| | | | | $R4K^{1-166}$ | 34 | 10 | $1.30 \times 10^{-3}$ |
| | | | | $T6A^{1-166}$ | 87 | 29 | $5.84 \times 10^{-7}$ |
| | | | | $T6D^{1-166}$ | 0 | 110 | $1.10 \times 10^{-78}$ |
| | | | | $T6E^{1-166}$ | 4 | 138 | $6.35 \times 10^{-85}$ |
| | | | | $R7A^{1-166}$ | 0 | 122 | $2.71 \times 10^{-84}$ |
| | | | | $R7K^{1-166}$ | 26 | 7 | $7.85 \times 10^{-3}$ |
| | | | | $N8A^{1-166}$ | 89 | 5 | 1 |
| | | | | $S9A^{1-166}$ | 21 | 3 | 0.21 |
| | | | | $S9D^{1-166}$ | 12 | 95 | $1.19 \times 10^{-57}$ |
| | | | | $S9E^{1-166}$ | 29 | 94 | $7.07 \times 10^{-47}$ |

**Table S1.** Con't.

| Figure | Construct 1 | N1 (ON CHR) | N1 (OFF CHR +CC) | Construct 2 | N2 (ON CHR) | N2 (OFF CHR +CC) | Fisher's Exact Test p-value |
|---|---|---|---|---|---|---|---|
| Figure S7 | $WT^{1-110}$ | 162 | 10 | $R4A^{1-110}$ | 50 | 10 | 0.02 |
| | | | | $T6A^{1-110}$ | 47 | 14 | $4.26 \times 10^{-4}$ |
| | | | | $T6D^{1-110}$ | 21 | 9 | $3.60 \times 10^{-4}$ |
| | | | | $T6E^{1-110}$ | 16 | 10 | $2.13 \times 10^{-5}$ |
| | | | | $R7A^{1-110}$ | 43 | 18 | $6.21 \times 10^{-6}$ |
| | | | | $N8A^{1-110}$ | 27 | 1 | 1 |
| | | | | $S9A^{1-110}$ | 43 | 1 | 0.47 |
| | | | | $S9D^{1-110}$ | 45 | 7 | 0.08 |
| | | | | $S9E^{1-110}$ | 43 | 4 | 0.51 |
| Figure S8A | $WT^{1-433}$ | 308 | 193 | $R40A^{1-433}$ | 1 | 64 | $3.45 \times 10^{-23}$ |
| | | | | $R42A^{1-433}$ | 0 | 88 | $1.10 \times 10^{-32}$ |
| | | | | $L45A^{1-433}$ | 33 | 10 | 0.02 |
| | | | | $\Delta DB^{1-433}$ | 62 | 53 | 0.14 |
| | | | | $\Delta DB/K42A^{1-433}$ | 3 | 29 | $3.70 \times 10^{-9}$ |
| | $R42A^{1-433}$ | 0 | 88 | $R40A^{1-433}$ | 1 | 64 | 0.42 |
| | | | | $\Delta DB^{1-433}$ | 62 | 53 | $2.08 \times 10^{-20}$ |
| | $\Delta DB^{1-433}$ | 62 | 53 | $\Delta DB/K42A^{1-433}$ | 3 | 29 | $4.42 \times 10^{-6}$ |
| Figure S8B | $WT^{1-63}$ | 99 | 5 | $R42A^{1-63}$ | 73 | 8 | 0.25 |
| | | | | $\Delta 3\text{-}8/R42A^{1-63}$ | 0 | 107 | $7.59 \times 10^{-55}$ |
| | $WT^{1-110}$ | 162 | 10 | $R42A^{1-110}$ | 101 | 10 | 0.35 |
| | | | | $\Delta 3\text{-}8/R42A^{1-110}$ | 0 | 121 | $3.32 \times 10^{-71}$ |
| | $WT^{21-110}$ | 56 | 18 | $R42A^{21-110}$ | 0 | 63 | $6.66 \times 10^{-23}$ |
