# Supplementary material for: Determinants of Human Cyclin B1 Association with Mitotic Chromosomes
Source: PLoS One. 2013 Mar 11;8(3):e59169. doi: 10.1371/journal.pone.0059169 (PMC3594322; doi:10.1371/journal.pone.0059169)
Supplement: Table S2 — Wilcoxon exact test p-values for quantitative analysis. (PDF) [file pone.0059169.s010.pdf]

Table S2. Wilcoxon exact test p-values for quantitative analysis. CER = Chromosome Enrichment Ratio (defined as the mean fluorescence intensity of the chromosomal region divided by the mean fluorescence intensity of the whole cell). SD = standard deviation. Vs. = the construct used for two-way comparison in the statistical analysis.

Figure	Construct	N=	Mean CER	SD	Vs.	Wilcoxon Exact Test P-value
Figure 2B	WT^{1-433}	10	1.87	0.20		
	GFP	10	1.37	0.09	WT^{1-433}	1.08×10^{-5}
	WT^{1-15}	11	1.39	0.10		5.67×10^{-6}
	WT^{1-20}	11	1.55	0.12		1.08×10^{-4}
	WT^{1-41}	8	1.97	0.31		0.57
	WT^{1-63}	8	2.45	0.25		1.83×10^{-4}
	WT^{1-110}	13	2.34	0.21		2.10×10^{-5}
	WT^{1-166}	14	2.14	0.26		7.25×10^{-3}
Figure 2D	WT^{21-433}	11	1.35	0.14	WT^{1-433}	5.67×10^{-6}
	WT^{42-433}	10	1.20	0.10		1.08×10^{-5}
	WT^{21-166}	10	1.66	0.12	WT^{1-166}	4.08×10^{-6}
	WT^{42-166}	10	1.37	0.13		1.02×10^{-6}
	WT^{21-110}	10	1.81	0.15	WT^{1-110}	1.22×10^{-5}
	WT^{42-110}	11	1.39	0.11		8.01×10^{-7}
Figure 3C	$\Delta 3\text{-}8^{1-433}$	10	1.51	0.15	WT^{1-433}	2.17×10^{-5}
	$R4A^{1-433}$	10	1.47	0.11		1.08×10^{-5}
	$T6A^{1-433}$	8	1.51	0.12		9.14×10^{-5}
	$T6D^{1-433}$	8	1.41	0.14		4.57×10^{-5}
	$R7A^{1-433}$	10	1.45	0.09		1.08×10^{-5}
	$N8A^{1-433}$	8	1.88	0.19		0.97
	$S9A^{1-433}$	9	2.01	0.21		0.16
	$S9D^{1-433}$	9	1.44	0.09		2.17×10^{-5}
	$E14A^{1-433}$	10	1.82	0.11		0.97

Table S2. Con't.

Figure	Construct	N=	Mean CER	SD	Vs.	Wilcoxon Exact Test P-value
Figure 4B	$\Delta 3\text{-}8^{1\text{-}166}$	14	1.54	0.29	$WT^{1\text{-}166}$	1.86×10^{-5}
	$\Delta 3\text{-}8^{1\text{-}110}$	11	1.70	0.21	$WT^{1\text{-}110}$	5.70×10^{-5}
	$\Delta 3\text{-}8^{1\text{-}63}$	8	1.83	0.29	$WT^{1\text{-}63}$	1.86×10^{-3}
	$\Delta 3\text{-}8^{1\text{-}41}$	10	1.42	0.10	$WT^{1\text{-}41}$	4.57×10^{-5}
Figure 5B	$R40A^{1\text{-}433}$	10	1.53	0.11	$WT^{1\text{-}433}$	2.17×10^{-5}
	$R42A^{1\text{-}433}$	10	1.61	0.08		3.25×10^{-4}
	$L45A^{1\text{-}433}$	12	1.82	0.16		0.50
	$\Delta DB^{1\text{-}433}$	9	1.85	0.20		0.84
	$\Delta DB/K42A^{1\text{-}433}$	14	1.54	0.19		1.15×10^{-3}
Figure 5D	$R42A^{1\text{-}63}$	13	2.09	0.25	$WT^{1\text{-}63}$	5.96×10^{-3}
	$\Delta 3\text{-}8/R42A^{1\text{-}63}$	13	1.39	0.15		9.83×10^{-6}
	$R42A^{1\text{-}110}$	11	2.18	0.17	$WT^{1\text{-}110}$	0.04
	$\Delta 3\text{-}8/R42A^{1\text{-}110}$	12	1.48	0.14		3.85×10^{-7}
	$R42A^{21\text{-}110}$	9	1.50	0.16	$WT^{21\text{-}110}$	2.60×10^{-4}
